# Computational Modeling of Fructose Metabolism and Development in NAFLD

**DOI:** 10.3389/fbioe.2020.00762

**Published:** 2020-07-22

**Authors:** Yunjie Liao, Nathan A. Davies, I. David L. Bogle

**Affiliations:** ^1^Department of Chemical Engineering, Center for Process Systems Engineering, University College London, London, United Kingdom; ^2^Division of Medicine, Institute for Liver and Digestive Health, University College London, London, United Kingdom

**Keywords:** fructose metabolism, NAFLD, computational modeling, triglyceride, systems biology

## Abstract

Non-alcohol fatty liver disease (NAFLD) is a common disorder that has increased in prevalence 20-fold over the last three decades. It covers a spectrum of conditions resulting from excess lipid accumulation in the liver without alcohol abuse. Among all the risk factors, over-consumption of fructose has been repeatedly reported in both clinical and experimental studies to be highly associated with the development of NAFLD. However, studying *in vivo* systems is complicated, time consuming and expensive. A detailed kinetic model of fructose metabolism was constructed to investigate the metabolic mechanisms whereby fructose consumption can induce dyslipidaemia associated with NAFLD and to explore whether the pathological conditions can be reversed during the early stages of disease. The model contains biochemical components and reactions identified from the literature, including ~120 parameters, 25 variables, and 25 first order differential equations. Three scenarios were presented to demonstrate the behavior of the model. Scenario one predicts the acute effects of a change in carbohydrate input in lipid profiles. The results present progressive triglyceride accumulations in the liver and plasma for three diets. The rate of accumulation was greater in the fructose diet than that of the mixed or glucose only models. Scenario two explores the variability of metabolic reaction rate within the general population. Sensitivity analysis reveals that hepatic triglyceride concentration is most sensitive to the rate constant of pyruvate kinase and fructokinase. Scenario three tests the effect of one specific inhibitor that might be potentially administered. The simulations of fructokinase suppression provide a good model for potentially reversing simple steatosis induced by high fructose consumption, which can be corroborated by experimental studies. The predictions in these three scenarios suggest that the model is robust and it has sufficient detail to present the kinetic relationship between fructose and lipid in the liver.

## Introduction

Non-alcoholic fatty liver disease (NAFLD) is the most common chronic liver dysfunction worldwide (Miele et al., 2009). It covers a spectrum of conditions resulting from excess lipid accumulation in the liver without excessive alcohol consumption. The pathologic manifestations of NAFLD develop from simple steatosis (intrahepatic lipid deposition) to non-alcoholic steatohepatitis (NASH), an advanced stage that combines steatosis with inflammation. NASH can then further progress to fibrosis (excess fibrous connective tissues) and cirrhosis (a late stage of scarring), and potentially to hepatocellular carcinoma (Ouyang et al., 2008; Cohen et al., 2011). Among these conditions, steatosis and NASH are reversible, while fibrosis and cirrhosis are often considered irreversible (Maldonado et al., 2018). Over the last three decades NAFLD has increased in its prevalence 20-fold, mainly linked with obesity, diabetes, and many other metabolic disorders. Currently it is globally affecting 20–30% of the general population (Nomura and Yamanouchi, 2012; Younossi et al., 2016). It has been estimated that between 2016 and 2030, NAFLD levels would continue to grow at a steady rate of up to ~30% (Estes et al., 2018). However, the molecular mechanisms causing NAFLD are multifactorial and remain poorly understood, which results in the absence of effective therapeutic interventions.

Although overnutrition and a sedentary lifestyle have often been blamed as the cause of NAFLD, recent clinical and experimental studies repeatedly suggest that the climbing consumption of fructose may also be an important factor (Jensen et al., 2018). Fructose, along with glucose and galactose, is one of three primary dietary monosaccharides. Between 1900 and 1950, ~20 g fructose (5% of total energy) was consumed in the daily meals, mainly from fruits and honey (Douard and Ferraris, 2008). Nowadays, fructose has become a ubiquitous ingredient that accounts for a large proportion of energy intake (approaching 15–25% of total energy) (Softic et al., 2016; Jensen et al., 2018). A 30% increase in total fructose consumption has been observed in recent decades (Ventura et al., 2011). The average fructose intake for the whole population in America has been reported as 49 g/day in 2004 (Douard and Ferraris, 2013) and 54.7 g/day in 2008 (Vos and Lavine, 2013). For the age groups 15–18 and 19–22, a reported total of 75 g fructose is consumed per day (Douard and Ferraris, 2013). Refined and processed fructose is responsible for this dramatic rise. Sucrose and high fructose corn syrup (HFCS) have become the main sources of fructose consumption, with a fructose/glucose ratio of 50/50 and 55/45, respectively (Ventura et al., 2011; Jensen et al., 2018). HFCS, a key component of sugar sweetened beverages, has been considered as an inexpensive substitute for other simple sugars in the food industry, accounting for 40% of all added sugars (Bray et al., 2004). Recently, this sweetener has been targeted by public health campaigns (e.g., sugar reduction programme in UK) and with a sugar tax levy in several countries (Jones, 2016; Briggs et al., 2017; Hashem et al., 2019).

Despite the fact that historically fructose was proposed as a beneficial sweetener and recommended for the obese and for patients with diabetes, high-fructose intake has been reported to be associated with a series of health issues such as metabolic syndrome, obesity, type 2 diabetes, and NAFLD (Basaranoglu et al., 2013). It is proposed that fructose is strongly associated with these chronic health issues due to its unique and distinct metabolic pathways which exclusively take place within the liver. It is known that, in contrast to glucose, fructose initiates its metabolism via the enzyme fructokinase after uptake by liver cells (hepatocytes), bypassing the crucial rate-limiting step of glycolysis and delivering abundant high energy substrates for subsequent metabolism (Ouyang et al., 2008). Growing evidence suggests that a high-fructose diet contributes to the enhancement of *de novo* lipogenesis, decreasing β-oxidation as well as increasing plasma levels of both triglyceride and very-low-density lipoprotein (VLDL) (Koo et al., 2008; Lim et al., 2010). Consequently, these metabolic effects would lead to hepatic lipid accumulation, insulin resistance and an increased inflammatory response which in turn contributes to NAFLD (Duarte et al., 2019). However, it is still controversial as to whether and how dietary fructose makes a unique contribution to the development of NAFLD in humans. The primary purpose of this paper is to investigate the effect of dietary fructose on hepatic energy metabolism and the consequences of its over-consumption in the development of NAFLD using models of known metabolic functions.

Studying *in vivo* systems is complicated, time consuming and expensive. A computational model based upon a systems biology approach is an attractive option to acquire a more comprehensive insight into the potential pathophysiological mechanisms involved.

With the benefit of extensive studies in modeling human metabolism over the last 20 years and with a systems biology approach called human genome-scale metabolic networks (GEMNs), reconstructions have been introduced. Three well-known reconstructed human metabolic networks have been established to incorporate complex metabolic pathways and biochemical reactions in humans (Duarte et al., 2007; Ma et al., 2007; Gille et al., 2010). Among these, the model “HepatoNet1” developed by Gille et al. (2010) mainly examines liver function at a system scale. It contains 777 components and over 2,500 reactions in order to explore ammonia detoxification rates and the synthesis of bile acids under starvation conditions. However, the major limitation of these stoichiometry-based reconstructed models is that their static predictions failed to represent the dynamic flows of metabolic reactions.

Applying a kinetic model to study liver energy metabolism has been investigated since the 1970s. The first model of metabolic regulation was introduced by Garfinkel (1971), which listed 34 dynamic chemical expressions to conduct the TCA cycle simulation. In recent decades a majority of hepatic models are focusing on the regulation of glucose homeostasis. Relevant *in silico* simulations have been reported, including glucose absorption and transportation, hormonal regulation, zonation effects, and the relationship between glucose intake and high-intensity exercise, lipid metabolism in addition to metabolic diseases (Chalhoub et al., 2007a,b; Hetherington et al., 2012; König et al., 2012; Sumner et al., 2012; Ashworth et al., 2016; Naftalin, 2016; Noorman et al., 2019). Irrespective of the fact that abundant studies have highlighted the important role fructose metabolism plays in metabolic diseases, only a few models have placed emphasis on the fructose metabolism and none of these have reflected the potentially dynamic changes (Allen and Musante, 2017, 2018; Maldonado et al., 2018).

Therefore, here we present a detailed kinetic model of fructose metabolism to investigate the metabolic mechanism whereby fructose consumption can induce changes to lipid metabolism associated with NAFLD, and to explore whether the pathological conditions can be reversed during the early stages of disease.

## Methods

### Model Description

**Figure 1:**
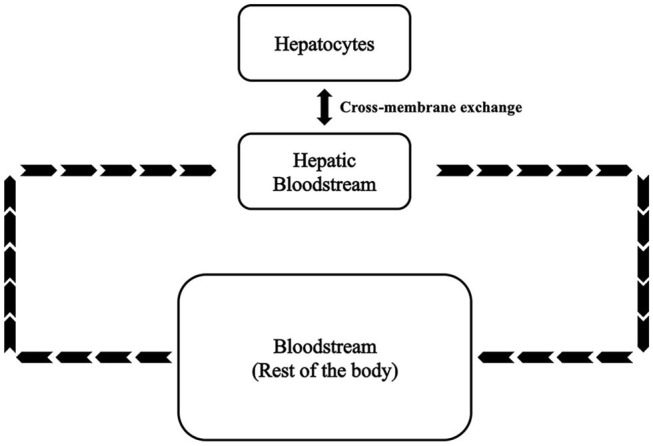
Basic framework of the fructose metabolism modeling.

A kinetic model of the fructose metabolism was developed based on modified Michaelis-Menten and Hill equations. This model comprises ~120 parameters, 25 variables, and 25 first order differential equations. As shown in Figure 1, variables and equations were divided into three sections representing hepatocytes (SH), hepatic bloodstream (SHB), and bloodstream of the rest of the body (SBC). The model parameters were determined and refined by comparison with values reported in the literature (see Table S1). Hepatic fatty acids (FA) and hepatic triglycerides (TG) are selected to be the major outputs in this model as they are the most important indices reflecting lipid accumulation in the liver. Plasma free fatty acids (FFA) and plasma triglycerides are also predicted as they are the most directly measurable matching indices recorded in clinical and experimental data. The equations are reported in this section.

### Hepatocytes–Fructose Metabolism

Since the metabolic activities of fructose mainly take place in the liver parenchyma, hepatocyte metabolism is the primary focus in this paper. As mentioned above, the most common assumption is to link fructose with NAFLD due to its unique metabolic processes. As fructokinase (also known as ketohexokinase, KHK), aldolase B, and triokinase are three specialized enzymes for fructose metabolism, the chemical reactions related to these three enzymes were first included to initiate the model construction. Substantial evidence leads to the proposition that high fructose consumption is attributable to enhancing *de novo* lipogenesis, suppressing β-oxidation and facilitating triglyceride synthesis (Koo et al., 2008; Lim et al., 2010; Tappy and Lê, 2010; Nomura and Yamanouchi, 2012). The model therefore was developed to incorporate these pathways. However, not every single component in the liver metabolism has been included. Pyruvate, acetyl-CoA, fatty acids and triglycerides were selected as they are identified as the most common intermediates and ultimate metabolites within the carbohydrate metabolic process associated with lipid deposition (Mayes, 1993; Sun and Empie, 2012; Laughlin, 2014). Also, they are considered to be the key components and they are assessable in clinical experiments, which allows the related parameters be tuned and validated during model development. Indeed, the reactions between these key metabolites in the human body are more complicated than that which is presented in the model. However, rate-determining enzymes among the biochemical processes were selected to simplify the reactions yet provide adequate details to represent realistic reaction rates.

**Figure 2:**
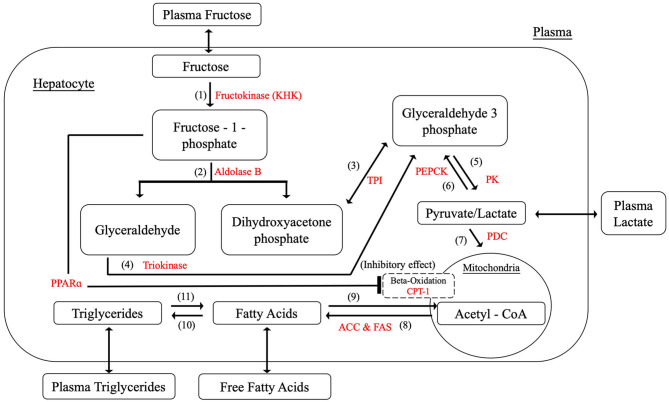
Hepatic fructose metabolism.

As a result, Figure 2 summarizes the biochemical components and reactions identified in the literature that are constructed within the model, including fructolysis, *de novo* lipogenesis (DNL), beta-oxidation, and triglyceride synthesis. Table 1 presents the rate equations for the hepatic variables used in this section.

**Table 1:** The rate equations for the hepatic variables in section Hepatocytes (SH).

**Hepatic variables**	**Abbreviation**	**Rate equations**
Fructose	Fru	$\frac{dFru}{dt}=T_{Fru}-{\mathbb{R}}_{KHK}$
Fructose-1-Phosphate	F1P	$\frac{dF1P}{dt}={\mathbb{R}}_{KHK}-{\mathbb{R}}_{ald\text{B}}$
Dihydroxyacetone phosphate	DHAP	$\frac{dDHAP}{dt}={\mathbb{R}}_{ald\text{B}}-{\mathbb{R}}_{TPI_{DHAP}}+{\mathbb{R}}_{TPI_{GA3P}}$
Glyceraldehyde	GA	$\frac{dGA}{dt}={\mathbb{R}}_{ald\text{B}}-{\mathbb{R}}_{Tri}$
Glyceradehyde-3-phosphate	GA3P	$\frac{dGA3P}{dt}={\mathbb{R}}_{TPI_{DHAP}}-{\mathbb{R}}_{TPI_{GA3P}}+{\mathbb{R}}_{Tri}-{\mathbb{R}}_{PK}+{\mathbb{R}}_{PEPCK}$
Pyruvate/lactate	Pyr	$\frac{dPyr}{dt}={T_{Lac}+\mathbb{R}}_{PK}-{\mathbb{R}}_{PDC}-{\mathbb{R}}_{PEPCK}$
Acetyl-CoA	ACoA	$\frac{dACoA}{dt}={\mathbb{R}}_{PDC}-{8\;\mathbb{R}}_{FAS}+{8\;\mathbb{R}}_{boxi}$
Fatty acids (palmitate)	FA	$\frac{dFA}{dt}={T_{FFA}+\mathbb{R}}_{FAS}-{\mathbb{R}}_{boxi}-{3\;\mathbb{R}}_{TGS}+{3\;\mathbb{R}}_{Lply}$
Triglycerides	TG	$\frac{dTG}{dt}={T_{TG}+\mathbb{R}}_{TGS}-{\;\mathbb{R}}_{Lply}$

#### The Distinctive Fructose Metabolic Pathways

The most significant distinction between glucose metabolism and fructose metabolism is their phosphorylation processes. After entering the hepatocyte, dietary fructose is swiftly phosphorylated by KHK to produce fructose-1-phosphate which bypasses the key rate-controlling regulatory enzyme (phosphofructokinase) of glycolysis in the glucose metabolism. Fructose-1-phosphate is then converted to dihydroxyacetone-phosphate (DHAP) and glyceraldehyde (GA) by aldolase B, providing intermediates for further glycolysis processes. Triokinase, the third essential enzyme, functions by phosphorylating GA to form glyceraldehyde-3-phosphate (GA3P), which also produces intermediates for subsequent reactions. The pathways of glucose and fructose metabolism then merge at the triose phosphate stage (as GA3P) and become the same from this point on (Havel, 2005; Rutledge and Adeli, 2007; Ouyang et al., 2008; Laughlin, 2014). Key enzymes and detailed reactions were demonstrated as follows. The corresponding metabolic functions are listed in Table 2.

(1) Hepatic fructokinase (KHK)

**Table 2:** The processes of metabolic reactions and rate functions in the fructose model.

	**Key enzymes/reactions**	**Abbreviation**	**Rate functions**
(1)	Fructokinase	KHK	${\mathbb{R}}_{KHK}=V_{KHK}*\frac{Fru^{nFru}}{{Km_{KHK}}^{nFru}+Fru^{nFru}}*\frac{ATP^{nATP}}{{Km_{ATP}}^{nATP}+ATP^{nATP}}$
(2)	Aldolase B	aldB	${\mathbb{R}}_{aldB}=V_{aldB}*\frac{{F1P}^{nF1P}}{{Km_{F1P}}^{nF1P}+{F1P}^{nF1P}}$
(3)	Triose phosphate isomerase	TPI	$\begin{array}{l} {\mathbb{R}}_{TPI\text{\_}DHAP}=V_{TPI\text{\_}DHAP}*\frac{DHAP^{nDHAP}}{{Km_{DHAP}}^{nDHAP}+DHAP^{nDHAP}}\\ {\mathbb{R}}_{TPI\text{\_}GA3P}=V_{TPI\text{\_}GA3P}*\frac{{GA3P}^{nGA3P}}{{Km_{TPIGA3P}}^{nGA3P}+{GA3P}^{nGA3P}} \end{array}$
(4)	Triokinase	Tri	${\mathbb{R}}_{Tri}=V_{Tri}*\frac{GA^{nGA}}{{Km_{GA}}^{nGA}+GA^{nGA}}*\frac{{ATP_{Mg^{2-}}}^{nATP_{Mg^{2-}}}}{{Km_{ATP_{Mg^{2-}}}}^{nATP_{Mg^{2-}}}+{ATP_{Mg^{2-}}}^{nATP_{Mg^{2-}}}}*\left(1-\beta_{ATP}\frac{ATP}{K_{i}^{ATP}+ATP}\right)\left(1-\beta_{ADP}\frac{ADP}{K_{i}^{ADP}+ADP}\right)$
(5)	Pyruvate kinase	PK	${\mathbb{R}}_{PK}=V_{PK}*\frac{{GA3P}^{nGA3P}}{{Km_{GA3P}}^{nGA3P}+{GA3P}^{nGA3P}}*\frac{ADP^{nADPpk}}{{Km_{ADPpk}}^{nADPpk}+ADP^{nADPpk}}*\left(1-\beta_{ACoA-PK}\frac{ACoA}{K_{i}^{ACoA-PK}+ACoA}\right)$
(6)	Phosphoenolpyruvate carboxykinase	PEPCK	${\mathbb{R}}_{PEPCK}=V_{PEPCK}*\frac{Pyr}{K_{m}^{PEPCK}+Pyr}*\frac{ATP}{K_{m}^{ATPpepck}+ATP}*\frac{GTP}{K_{m}^{GTP}+GTP}$
(7)	Pyruvate oxidation	PDC	${\mathbb{R}}_{PDC}=V_{PDC}*\frac{Pyr}{K_{m}^{Pyr}+Pyr}*\left(1-\beta_{ACoA-PDC}\frac{ACoA}{ACoA+k_{i}^{CoA-pyr}}\right)$
(8)	Fatty acid synthesis	FAS	${\mathbb{R}}_{FAS}{=V}_{FAS}*\frac{ACoA}{K_{m}^{ACoA}+ACoA}*\frac{ATP}{K_{m}^{ATPfas}+ATP}*\left(1-\beta_{FA}\frac{FA}{FA+k_{i}^{FA-inhib}}\right)$
(9)	Beta-oxidation	boxi	${\mathbb{R}}_{boxi}{=V}_{boxi}*\frac{FA}{K_{m}^{boxi}+FA}*\frac{ATP}{K_{m}^{ATPboxi}+ATP}*\left(1-\beta_{boxi}\frac{ACoA}{ACoA+k_{i}^{CoA-boxi}}\right)*\left(1-\beta_{PPAR\alpha }\frac{F1P}{F1P+k_{i}^{F1P-inhib}}\right)$
(10)	Triglyceride synthesis	TGS	${{\mathbb{R}}_{TGS}=V}_{TGS}*\frac{FA}{K_{m}^{FA}+FA}*\frac{GA3P}{K_{m}^{TGSGA3P}+GA3P}$
(11)	Lipolysis	Lply	${\mathbb{R}}_{Lply}=V_{Lply}*\frac{TG}{K_{m}^{TG}+TG}$

$$\begin{array}{l} Fructose+ATP\overset{KHK}{\to }Fructose-1-phosphate+ADP \end{array}$$

Hepatic fructokinase (KHK, EC 2.7.1.3), one of the three characteristic enzymes in human fructose metabolism, converts fructose into fructose-1-phosphate (F1P) by transferring one phosphate group from adenosine triphosphate (ATP). In contrast to glucose phosphorylation, there is no feedback inhibition for fructose which indicates that the activity of KHK is essentially free of regulatory control. Consequently, when sufficient fructose is available a significant amount of F1P enters subsequent metabolic reactions. Also, since the Michaelis constant (Km) of KHK is lower than glucokinase, it has been shown that KHK is effectively 10-times faster than glucokinase in substrate phosphorylation (Patel et al., 2015). In terms of energy transport, even though guanosine triphosphate (GTP) can also be utilized in a similar way to ATP for this initial phosphorylation reaction, it is only responsible for a minor proportion of the total process and the effect of GTP can be ignored in this equation.

(2) Aldolase B

$$\begin{array}{l} Fructose-1-phosphate\overset{AldolaseB}{\to }Dihydroxyacetone\\ phosphate+Glyceraldehyde \end{array}$$

After phosphorylation, fructose-1-phosphate (F1P) undergoes further breakdown into two three-carbon components, namely dihydroxyacetone phosphate (DHAP) and glyceraldehyde (GA) by aldolase B. Aldolase B (E.C.4.1.2.13) is a liver-specific aldolase which can be considered the rate-limiting enzyme of hepatic fructose metabolism. Since little is known about the mechanism of aldolase B regulation, no strong allosteric control has yet been identified for this enzyme.

(3) Triose Phosphate Isomerase (TPI)

$$\begin{array}{l} Dihydroxyacetone\;phosphate\overset{TPI}{\Leftrightarrow }GA3P \end{array}$$

DHAP is isomerised to glyceradehyde-3-phosphate (GA3P) by triose phosphate isomerase (TPI) (E.C.5.3.1.1) rapidly and reversibly.

(4) Triokinase

$$\begin{array}{l} Glyceraldehyde+ATP\;\overset{Triokinase}{\to }GA3P+ADP \end{array}$$

The primary pathway for the GA metabolism is through GA3P catalyzed by triokinase (E.C.2.7.1.28). This reaction requires one phosphate molecule from ATP, releasing adenosine diphosphate (ADP). The activity of triokinase is allosterically activated by ATP-Mg^−2^ and inhibited by both ATP and ADP, suggesting that this hepatic triokinase is regulated by the phosphorylation potential in the cytoplasm. Under normal conditions triokinase is fully activated.

(5) Pyruvate Kinase (PK)

$$\begin{array}{l} GA3P+2ADP+2Pi\;\overset{PK}{\to }\;Pyruvate/Lactate+2ATP \end{array}$$

As pyruvate can be converted to lactate swiftly and reversibly, only one variable is used to denote this in the model. The pathways for glucose and fructose metabolism merge at the triose phosphate stage and become the same from this point onwards. GA3P is broken down to pyruvate relying on a series of enzyme reactions. The rate limiting enzyme in this process is pyruvate kinase (PK; E.C.2.7.1.40). Here we simplify the whole six-step conversion of GA3P to pyruvate by using PK. The phosphate in the GA3P and an additional free inorganic phosphate are combined with ADP molecules to produce two ATP molecules in this process. It should be noted that there are two GA3P molecules generated from one fructose molecule in the previous metabolic step, four ATP and two pyruvate molecules are therefore produced in the current reaction. Pyruvate kinase is allosterically controlled by acetyl-CoA.

(6) Phosphoenolpyruvate carboxykinase (PEPCK)

$$\begin{array}{l} Pyruvate/Lactate+2ATP+GTP\overset{PEPCK}{\to }\;GA3P+2ADP\\ \;+GDP+2Pi \end{array}$$

Phosphoenolpyruvate carboxykinase (PEPCK) (E.C.4.1.1.32) is rate limiting in the conversion from pyruvate to GA3P, consuming two ATPs and one guanosine triphosphate (GTP). There is no identified allosteric regulation for PEPCK while numerous metabolites, such as insulin and fatty acids, are able to stimulate its production. Over-expression of PEPCK is believed to be associated with high production of glucose and the development of type 2 diabetes (Beale et al., 2007).

(7) Pyruvate Dehydrogenase Complex (PDC)

$$\begin{array}{l} Pyruvate/Lactate+NAD^{+}\overset{PDC}{\to }Acetyl-CoA+NADH \end{array}$$

Pyruvate oxidation is regulated by the pyruvate dehydrogenase complex (PDC). This complex contains three enzymes that catalyze the conversion of pyruvate to acetyl-CoA. PDC is allosterically inhibited in a feedback mechanism by acetyl-CoA to prevent its over-production, which could result in mitochondrial stress.

(8) Fatty acid synthesis

$$\begin{array}{l} 8\;Acetyl-CoA+7\;ATP\;\overset{ACC\&FAS}{\to }Fatty\;acid\;\left(Palmitate\right)\\ \;+7\;ADP+7Pi \end{array}$$

Lipogenesis describes the process of fatty acid synthesis and triglyceride synthesis. With the mediation of acetyl-CoA carboxylase (ACC) (E.C.6.4.1.2) and fatty acid synthase (FAS) (E.C.2.3.1.85), acetyl-CoA is converted into malonyl-CoA. Malonyl-CoA provides the two-carbon structure for producing both short and long chain fatty acids. There are two isoforms of ACC found in the hepatic metabolism as ACC1 contributes to lipogenesis and ACC2 to beta-oxidation. Palmitate (16:0), as the most common saturated fatty acid, has been chosen to represent fatty acids in this model for the purpose of simplification. Thus, eight acetyl-CoA molecules are consumed to synthesize one palmitate molecule. High concentrations of fatty acids are able to suppress this process allosterically.

(9) Beta-oxidation

$$\begin{array}{l} Fatty\;acid\;\left(Palmitate\right)+2ATP\overset{CPT-1\left(PPAR\alpha \right)}{\underset{}{\to }}\;8Acetyl-CoA\\ \;+AMP+ADP+3Pi \end{array}$$

Hepatic carnitine palmitoyltransferase I (CPT-1) (E.C.2.3.1.21) is the rate-controlling enzyme of beta-oxidation. The metabolic process breaks down fatty acids to generate acetyl-CoA (Lim et al., 2010). Since malonyl-CoA is the main inhibitor for CPT-1, it supresses beta-oxidation allosterically. To simplify the equation, the inhibitory effect of malonyl-CoA is substituted by acetyl-CoA as the pathway of acetyl-CoA to produce malonyl-CoA is unidirectional. By contrast, it has been discovered recently that peroxisome proliferator-activated receptors (PPARs) promote beta-oxidation by upregulating the expression of CPT1 (Kersten, 2014). However, this regulation can be prevented by the production of fructose-1-phosphate (Nomura and Yamanouchi, 2012). Therefore, fructose-1-phosphate is also considered to be an allosteric inhibitor in the process of beta-oxidation.

(10) Triglyceride Synthesis

$$\begin{array}{l} 3\;Fatty\;acids\;\left(Palmitate\right)+3\;ATP\;\overset{Glycerol-3-phosphate}{\underset{}{\to }}\\ Triglyceride+3AMP+7Pi \end{array}$$

During triglyceride synthesis, three fatty acid molecules and one glycerol backbone from glycerol-3-phosphate are combined to produce triglyceride under the influence of coenzyme A (CoA) and several acyltransferases. Glycerol-3-phosphate is denoted as GA3P due to the rapid exchange rate between these two molecules. Triglyceride synthesis is regulated by insulin and glucagon.

(11) Lipolysis

$$\begin{array}{l} Triglyceride\;\overset{Triacylglycerollipase}{\underset{}{\to }}Glyceraldehyde+\;3Fatty\;acids\;\\ \;\left(Palmitate\right) \end{array}$$

Three fatty acids are released when one triglyceride breaks down. The rate-determining enzyme during this process is triacylglycerol lipase (E.C.3.1.1.3). The concentration of hepatic triglyceride is regulated by insulin and glucagon under normal circumstances.

### Hepatic Bloodstream: Cross-Membrane Exchange

The hepatic blood flow is maintained by hepatic arteries, portal veins, central veins, and bile ducts which allow hepatocytes to be exposed to nutrients, hormones (insulin and glucagon), and oxygen (Hijmans et al., 2014). As applied in König et al. (2012) and Ashworth et al. (2016), an altered Michaelis–Menten equation is employed for cross-membrane exchange. For unidirectional transportation, the model considers the components in the Hepatic Bloodstream as substrates and the corresponding molecules in the hepatocytes as products. For bidirectional exchange the equation for transport (*T*) is:

$$\begin{array}{l} T_{SHB\to SH\;}=\frac{V_{max}\left(S_{SHB}-S_{SH}\right)}{K_{m}+\;S_{SHB}+S_{SH}} \end{array}$$

where the (section) Hepatic Bloodstream is denoted as SHB and (section) Hepatocytes denoted as SH.

The transportation and exchange rates of fructose, pyruvate/lactate, fatty acids, and triglyceride between SHB and SH are summarized in Table 3. The constant *R*_*HE*_ = 4 is used to represent the ratio of the total number of hepatocytes to the volume of the hepatic bloodstream as described in Ashworth et al. (2016). The rate equations in SHB are listed in **Table 5** combined with the rate equations for the section Bloodstream Circulation: Rest of the Body.

**Table 3:** The transport and exchange rates between Section Hepatic Bloodstream (SHB) and Section Hepatocytes (SH).

**Transport variables**	**Rate functions for cross-membrane transportation**
Fructose	$T_{Fru}=V_{GLUT2}^{pump}*\frac{Fru_{SHB}}{K_{m}^{GLUT2-pump}+Fru_{SHB}}+V_{GLUT2}^{ex}*\frac{Fru_{SHB}-Fru_{SH}}{K_{m}^{GLUT2-ex}+Fru_{SHB}+Fru_{SH}}+V_{GLUT5}^{pump}*\frac{Fru_{SHB}}{K_{m}^{GLUT5-pump}+Fru_{SHB}}+V_{GLUT5}^{ex}*\frac{Fru_{SHB}-Fru_{SH}}{K_{m}^{GLUT5-ex}+Fru_{SHB}+Fru_{SH}}$
Pyruvate/lactate [simplified from Ashworth et al. (2016)]	$T_{Pyr}=V_{Pyr}^{ex}*\frac{Pyr_{SHB}-Pyr_{SH}}{K_{m}^{Pyr-ex}+Pyr_{SHB}+Pyr_{SH}}$
Fatty acids (palmitate) [simplified from Ashworth et al. (2016)]	$T_{FA}=V_{FA}^{ex}*\frac{FA_{SHB}-FA_{SH}}{K_{m}^{FA-ex}+FA_{SHB}+FFA_{SH}}+V_{active}*\frac{FA_{SHB}}{\left(K_{m}^{active}+FA_{SHB}\right)}\left(1+\frac{Ins}{Ins_{ref}^{active}}\right)$
Triglyceride [simplified from Ashworth et al. (2016)]	$T_{TG}=V_{TG}^{ex}*\frac{\left(TG_{SHB}-\frac{TG_{SH}}{TG_{ref}}\right)}{K_{m}^{TG-ex}+TG_{SHB}+\frac{TG_{SH}}{TG_{ref}}}-\;V_{out}*\frac{TG_{SH}}{\left(K_{m}^{out}+TG_{SH}\right)}$

Fructose is absorbed from the gut lumen and transported across the brush border membrane into the hepatic portal vein via an energy-dependent process involving glucose transporter 5 (GLUT5) and glucose transporter 2 (GLUT2), in which GLUT 5 has a high specificity to fructose (Douard and Ferraris, 2008). After fructose uptake from the gut, plasma fructose is observed experimentally to rise only by micromolar levels, implying that hepatocytes have the capacity to uptake the majority of fructose during the first-pass through the liver (Tappy and Lê, 2010). Therefore, both GLUT2 and GLUT5 are included in the model where the Michaelis–Menten constant of GLUT2 has a lower value than that of GLUT5 as reported previously (Wright et al., 2012). In addition, GLUT 8 has also been mentioned in terms of hepatic fructose transportation (Manolescu et al., 2007; DeBosch et al., 2014). However, as the expression of GLUT8 is relatively low in mice in comparison to GLUT5 and GLUT2 even with high-fructose exposure, and the fact that the exact mechanism of this transporter in humans remains largely unknown, the effect of GLUT8 is considered negligible in the current model (Ferraris et al., 2018).

### Bloodstream Circulation-Rest of the Body

Since circulation of the bloodstream around the body takes ~1 min to complete, the rate of blood flow circulation is set to be $R_{BS}=1/60\approx 0.167\;s^{-1}$. Also, as the blood volume of the whole body and the liver are considered to be ~5 and 0.8 L, respectively, in an average person, the ratio of the rest of body to the liver *R*_*RL*_ is set as:

$$\begin{array}{l} R_{RL}=\frac{5-0.8}{0.8}\approx 5.25 \end{array}$$

(Arias et al., 1994; Davy and Seals, 1994; Critchley and Critchley, 1999; Eipel et al., 2010; Ashworth et al., 2016).

The equation representing the blood circulation (C) from (section) Hepatic Bloodstream (SHB) to (section) Bloodstream Circulation (SBC) is in the following form:

$$\begin{array}{l} C_{SBC\to SHB\;}=\frac{R_{BS}\;*\;\left(C_{SBC}{-C}_{SHB}\right)}{R_{RL}} \end{array}$$

### Dietary Inputs

After feeding, carbohydrates are processed in the digestive system and enter the hepatic portal system. According to the Dietary Guideline for Americans (2015–2020) (Health and Services, 2015), daily caloric intake of a healthy adult is in the range of 1,600–3,000 kcal, of which, 45–65% are derived from carbohydrates. Therefore, in this model, we set up the baseline to reflect a midpoint caloric consumption of 2,400 kcal per day and 50% of this energy source to be obtained from carbohydrates. A total amount of 300 g/day carbohydrates (4 kcal/g) was set to be taken up into the body from the diet. The remaining calorie intake would be comprised of proteins and fats. However, as the model has been set to examine the effects of carbohydrates in the liver, protein and fat inputs are not considered in this study.

**Figure 3:**
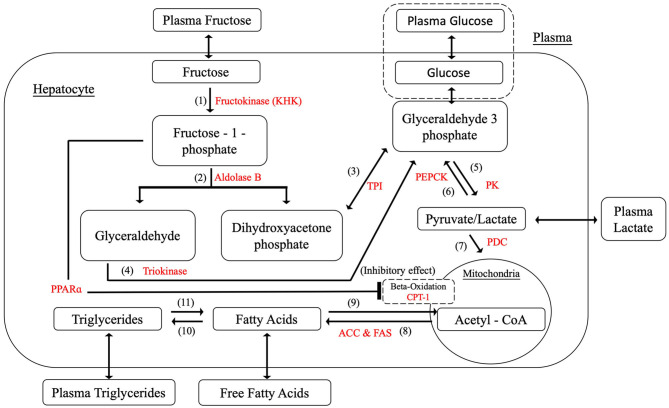
Hepatic fructose metabolism with glucose input.

After a mixed meal, dietary disaccharides and polysaccharides such as sucrose, HFCS and starches would be broken down into the various proportions of monosaccharides; hence, fructose and glucose as the principle simple sugars in the diet have been selected as the dietary inputs for the model. Figure 3 shows that glucose was added to the model as an alternative dietary input to fructose and relevant equations are simplified from model constructed by Ashworth et al. (2016), as presented in Table 4.

**Table 4:** The relevant equations of glucose feeding [simplified from Ashworth et al. (2016)].

**Hepatic variables**	**Rate equations**
Glucose (Glu)	$\frac{dGlu}{dt}=T_{Glu}-{\mathbb{R}}_{GK}+{\mathbb{R}}_{G6Pase}$
Glucose-6-phosphate (G6P)	$\frac{dG6P}{dt}={\mathbb{R}}_{GK}-{\mathbb{R}}_{G6Pase}+{\;\mathbb{R}}_{FBP}-{\mathbb{R}}_{PFK}$
**Key enzymes/reactions**	
**T**_**Glu**_ – Glucose transportation	$T_{Glu}=V_{GLUTG}^{pump}*\frac{Glu_{SHB}}{K_{m}^{GLUTG-pump}+Glu_{SHB}}+V_{GLUTG}^{ex}*\frac{Glu_{SHB}-Glu_{SH}}{K_{m}^{GLUTG-ex}+Glu_{SHB}+Glu_{SH}}$
Glucokinase (GK)	${\mathbb{R}}_{GK}=V_{GK}*\frac{Glu^{nGlu}}{{Km_{Glu}}^{nGlu}+Glu^{nGlu}}*\frac{ATP^{nATP}}{{Km_{ATPgk}}^{nATP}+ATP^{nATP}}*\left(1-\frac{G6P}{G6P+k_{i}^{G6P}}\right)$
Glucose-6-phosphatase (G6Pase)	${\mathbb{R}}_{G6Pase}=V_{G6Pase}*\frac{G6P}{K_{m}^{G6Pase}+G6P}$
Fructose-bisphosphatase (FBP)	${\mathbb{R}}_{FBP}=V_{FBP}*\frac{GA3P}{K_{m}^{FBP}+GA3P}$
Phosphofructokinase (PFK)	${\mathbb{R}}_{PFK}=V_{PFK}*\frac{G6P}{K_{m}^{PFK}+G6P}*\frac{ATP}{Km_{ATPpfk}+ATP}*\left(1-\frac{ATP}{ATP+k_{i}^{ATPfpk}}*\frac{ADP}{ADP+k_{i}^{ADPfpk}}\right)*\left(1-\beta_{PFK}\frac{GA3P}{GA3P+k_{i}^{GA3Ppfk}}\right)$

Periodic simulation of the carbohydrate intake (shown below) is based on spiked inputs adapted from Ashworth et al. (2016), which provides three meals a day with 4-h breaks.

$$\begin{array}{l} Dietary\;Input=v_{input}\;*\;\sin^{6}\left(\frac{pi}{4\left(hours\right)}\right) \end{array}$$

### Additional Settings

The settings for hormone regulation including insulin and glucagon secretion into the bloodstream (SBC) are based on the model constructed by Hetherington et al. (2012), while the hormonal equations in the liver blood flow (SHB) are simplified from the model built by Ashworth et al. (2016). In addition to the liver, the amount of each key variable (fructose, glucose, pyruvate/lactate, fatty acid, and triglyceride) generated (*USE*_*variable*_) and used (*UP*_*variable*_) by other body sections such as adipose tissues and muscle tissues are set to be the same as the equations described by Ashworth et al. (2016).

Overall, the final rate equations in both SHB and SBC are described in Table 5.

**Table 5:** The rate equations in both Section Hepatic Bloodstream (SHB) and Section Bloodstream Circulation (SBC).

**Exchanging variables**	**Rate equations in Section Hepatic Bloodstream (SHB)**
Fructose	$\frac{dFru_{SHB}}{dt}=-T_{Fru}*R_{HE}+R_{BS}*({Fru}_{SBC}-Fru_{SHB})$
Glucose [simplified from Ashworth et al. (2016)]	$\frac{dGlu_{SHB}}{dt}=-T_{Glu}*R_{HE}+R_{BS}*({Glu}_{SBC}-Glu_{SHB})$
Pyruvate/lactate [simplified from Ashworth et al. (2016)]	$\frac{dPyr_{SHB}}{dt}=-T_{Pyr}*R_{HE}+R_{BS}*({Pyr}_{SBC}-Pyr_{SHB})$
Fatty acids (palmitate) [simplified from Ashworth et al. (2016)]	$\frac{dFA_{SHB}}{dt}=-T_{FA}*R_{HE}+R_{BS}*({FA}_{SBC}-FA_{SHB})$
Triglyceride [simplified from Ashworth et al. (2016)]	$\frac{dTG_{SHB}}{dt}=-T_{TG}*R_{HE}+R_{BS}*({TG}_{SBC}-TG_{SHB})$
**Exchanging variables**	**Rate equations in Section Bloodstream Circulation (SBC)**
Fructose	$\frac{dFru_{SBC}}{dt}=Meal_{Fru}+C_{Fru}$
Glucose [simplified from Ashworth et al. (2016)]	$\frac{dGlu_{SBC}}{dt}=Meal_{Glu}+C_{Glu}-USE_{Glu}-UP_{FA}-UP_{TG}$
Pyruvate/lactate (simplified from Ashworth et al. (2016)]	$\frac{dPyr_{SBC}}{dt}=C_{Pyr}$
Fatty acids (palmitate) [simplified from Ashworth et al. (2016)]	$\frac{dFA_{SBC}}{dt}=C_{FA}-USE_{FA}+UP_{FA}$
Triglyceride [simplified from Ashworth et al. (2016)]	$\frac{dTG_{SBC}}{dt}=C_{TG}{-USE}_{TG}+UP_{TG}$

### Model Simulations

Simulations were generated using MATLAB_R2019a (MATLAB, RRID:SCR_001622). Function “ode45” was used to solve all the ordinary differential equations in parallel. The units of metabolite concentration and reaction rate are presented in micromoles/liter (μM/L) and micromoles/second (μM/s), respectively. The time lengths of the simulations have been set to run over a 12-h period incorporating three meals. The Matlab code is provided in Table S2.

## Results and Discussion

To demonstrate the behavior of model, three scenario simulations were conducted. The first predicts lipid concentrations resulting from different dietary consumptions, the second explores the variability of biochemical reaction kinetics rate within the general population, and the third tests the effect of one specific inhibitor of lipid metabolism contributing to NAFLD that might be potentially administered.

### Scenario One: The Effect of Changing Carbohydrate Intake on Lipid Accumulation

**Figure 4:**
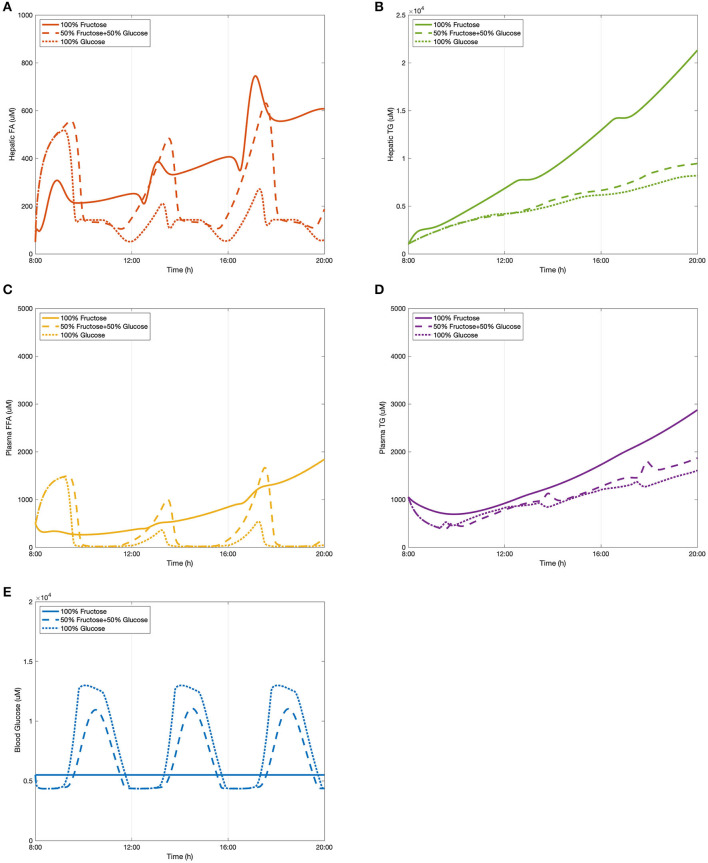
The change of lipid accumulation after different dietary intakes. **(A)** Hepatic FA, **(B)** hepatic TG, **(C)** plasma FFA, **(D)** plasma TG, and **(E)** blood glucose.

As described in section “Dietary Inputs,” a dietary setup was created in which 100 g of carbohydrates were consumed by healthy subjects for each meal (3/day). Here we tested the effect of three different diets on lipid deposition, including: a 100% fructose meal, a mixed meal (50:50 fructose and glucose), and a 100% glucose meal, representing two extreme conditions and one more realistic setting. The results of these simulations are presented in Figure 4. The levels of hepatic fatty acids (FA), triglycerides (TG), plasma free fatty acids (FFA), triglycerides, as well as blood glucose were predicted after three meals at 8:00, 12:00, and 16:00, respectively. The model takes approximately an hour for the initial transient phrase before establishing a set of consistent predictions.

As shown in Figure 4A, hepatic fatty acids started decreasing after the first meal and reached a peak at around 13:00 in the fructose feeding group and ~13:30 in both glucose and the mixed diet subjects. These decreasing patterns is because three fatty acids are required to generate one triglyceride molecule after meals during the triglyceride synthetic process. After the second meal, the subsequent peak time of fatty acid levels stimulated by all three diets was observed between 17:00 and 18:00, and again the fructose group took slightly less time to achieve the peak value. Even though the consumption of 50% glucose and 50% fructose resulted in the highest level of hepatic fatty acids for the first peak, a significantly higher concentration was induced by the fructose diet after the second meal. The glucose feeding group were found to have the lowest levels of fatty acids over the observation period. The concentration of hepatic FA in the mix-meal feeding group displayed stronger periodic behavior than in the other two extreme conditions. In terms of hepatic triglyceride (Figure 4B), the curves of the glucose group and the mix-carbohydrate group overlapped for several hours after breakfast. From around 13:00, the mix group started accumulating larger amounts of hepatic triglycerides than that of the glucose group. After consumption of the fructose-only diet, triglyceride concentration showed relatively dramatic increasing growth leading to the highest levels observed.

In addition to hepatic lipid concentrations, Figures 4C,D show the plasma levels of free fatty acids and triglycerides respectively. Similar to the periodic patterns of hepatic fatty acids, free fatty acids in the plasma declined over several hours before developing the first peak. For both mix-sugar feeding and glucose only groups, the levels of free fatty acids then returned to baseline after about 30 min. The second peaks of these two groups generated slightly higher values than the first peaks before again reducing to baseline. Regarding the 100% fructose model, this showed a smaller fluctuation than the other two diets and presented a flattened profile, and was not seen to drop back to the baseline level. Consistent with hepatic FA observations, plasma FFA peaked earlier in the fructose group than the others. Considering plasma triglyceride levels (Figure 4D), the fructose diet produced the highest concentrations throughout the model simulation. After the first meal, the glucose group contributed to greater triglyceride production than that induced by the mix group. However, this phenomenon shifted over during the 12:00–13:00 period where the mixed sugar group outgrew the glucose meal and continued increasing plasma triglyceride levels.

Blood glucose levels were also simulated after consuming different proportions of monosaccharides. A baseline normal blood glucose level was established within the model and the effects of the meals examined in addition to this. As presented in Figure 4E, the predicted peak values for each diet model throughout the period were equal in magnitude since the three meals were divided and induced equally. After pure fructose meals, blood glucose stayed relatively constant along the time period, while for the pure glucose group and the mix-sugar group, both of them caused dynamic periodic oscillations. It is apparent that blood glucose responded more strongly to the pure glucose diet than the mixed diet. As the models were considering healthy individuals, the effects of insulin in mediating cellular glucose uptake from the blood became more apparent once levels exceeded upper normal values (>1.2 mM), which can be observed as a blunting of the peak glucose levels in the figure.

Overall, it can be observed that it took ~5 h on average to digest dietary meals containing 100 g carbohydrates to subsequently achieve peak values in lipid profiles. Compared to fatty acids, triglycerides in the liver and plasma progressively accumulated. The rate of accumulation was greater in the fructose diet than that of the mixed or glucose only models. For both fatty acids and triglyceride concentrations, glucose meals were observed to result in the lowest levels over the study period. Additionally, for the 50/50 glucose/fructose model, the fatty acid curves fluctuated more dramatically in comparison to the other dietary inputs.

Clinical data from Abraha et al. (1998), Chong et al. (2007), and Stanhope et al. (2008) were used to compare the model predictions. The research carried out by Chong et al. (2007) recruited 14 healthy individuals to have one test meal either containing 0.75 g/kg body weight fructose or glucose. Plasma composition was recorded over 6 h to investigate the acute effect of high-carbohydrate diets. In the study by Stanhope et al. (2008), a larger sample size and a larger time scale were applied with more abundant dietary carbohydrate forms. A total of 34 subjects were provided with three meals with sucrose or HFCS drinks. For this study eight men also participated in a sub-study that included pure glucose and pure fructose consumption. Blood samples of all participants were collected over 24-h. In contrast to these two studies, Abraha et al. (1998) also included diabetic patients as subjects of an investigation to explore the effect of fructose on post-prandial lipid profiles. Six healthy individuals and six diabetic individuals were provided a test meal with a fructose-enriched drink or starch-enriched bread. Plasma metabolites for both groups were recorded for 6 h. These data were chosen as they covered varying dietary carbohydrate compositions in both healthy and diabetic subjects, which were considerably suitable for testing the compatibility of the constructed model. It should be noted that since diabetic and NAFLD patients are considered to have similar insulin responses when inducing high-carbohydrate meals, the clinical values measured in these diabetic subjects were regarded as reference indices. Also, as hepatic lipid levels are difficult to measure in clinical studies, only plasma lipid profiles were employed to make comparisons with the data from the selected studies.

Our model concurred with the findings from these three studies in the following ways. Model predictions and literature data took roughly the same time to process fructose metabolism. Specifically, the plasma FFA concentrations in the simulated data dropped for roughly 90 min before they rose to reach a peak after ~5 h. Also, consistent with results from these experimentally measured data, in healthy subjects the incremental plasma triglyceride concentration was higher after pure fructose meals than the other meal plans. The simulated results show that consumption of pure glucose can be attributed to the lowest triglyceride levels for the various diets, which is in keeping with the findings in Stanhope et al. (2008). Additionally, even though it is relatively difficult for computational models to make predictions matching the exact values of clinical measured data, the plasma triglyceride concentrations the model simulated here fitted within the range of 1,000–2,000 μmol for both glucose and mix-sugar feeding groups in the healthy subjects. Despite that pure fructose exposure produced values as high as 3,000 μmol plasma TG in the model predictions, this level was still lower than the plasma TG measured in the diabetic individuals after a fructose test meal (around 3,800 μmol). This result suggests that the model predictions are indicative of the normal range of plasma TG levels in a healthy population, even when considering two extreme conditions. Blood glucose predictions were also in agreement with the literature findings above. Furthermore, there are some discrepancies between the experimental figures and simulated numbers. We note that the peaks of plasma FFA induced by consumption of the mixed diet were higher than that of pure fructose diet. However, this inconsistency may be caused by the mechanism whereby fructose is able to enhance hepatic glucose uptake, hence indicating a synergistic effect of fructose and glucose in fatty acid synthesis.

### Scenario Two: The Effect of Varying Reaction Rate Constants on the Hepatic Metabolic Process

Rate constants indicate the maximum capacity of the enzymes in metabolic reactions, which are affected by numerous factors, e.g., age, diet, life style, and genetic predisposition. To explore the variability of these rate constants in the hepatic metabolic processes, an OAT (one at a time) sensitivity analysis was conducted by changing 11 key rate constants in the fructose pathway within the hepatocytes (SH). Since the normal range for healthy liver function can vary substantially between individuals, possibly by as much as 25% according to dye clearance measures designed to assess detoxification function (Vos et al., 2014), a mid-point 10% variation was applied to each rate constant to reflect the expected metabolic differences among healthy subjects. It is reasonable to choose this value as it is large enough to produce obvious changes on lipid levels that allow us to recognize the relative importance of metabolic reactions, but small enough to maintain within a healthy range. A 50/50 fructose/glucose diet was set as the standard input and the simulations were run for 12 h for acute effect consideration. Both hepatic and plasma concentrations of fatty acids and triglycerides were recorded as the end points for the analysis. The results are displayed in Table 6.

**Table 6:** Results for sensitivity analysis of rate constants in Section Hepatocytes (SH).

	**Key enzymes/reactions**	**Abbreviation**	**Δ** **Hepatic fatty acids (μM)**		**Δ** **Hepatic triglyceride (μM)**		**Δ** **Plasma fatty acids (μM)**		**Δ** **Plasma triglyceride (μM)**	
			**+10%**	**−10%**	**+10%**	**−10%**	**+10%**	**−10%**	**+10%**	**−10%**
(1)	Fructokinase	KHK	3.44	–5.07	281.15	–325.16	22.24	–25.85	42.43	–49.38
(2)	Aldolase B	aldB	–18.87	17.80	152.01	–211.55	–6.23	1.98	13.24	–22.06
(3)	Triose phosphate isomerase	TPI	0.00	0.00	0.78	–0.94	0.00	–0.01	0.18	–0.19
(4)	Triokinase	Tri	0.00	0.00	0.03	–0.05	0.00	0.00	0.01	–0.01
(5)	Pyruvate kinase	PK	25.27	–25.88	320.09	–357.91	38.59	–37.67	74.51	–83.11
(6)	Phosphoenolpyruvate carboxykinase	PEPCK	–16.05	18.33	–222.74	241.06	–22.97	27.00	–52.58	57.29
(7)	Pyruvate oxidation	PDC	15.61	–16.73	217.04	–244.18	22.66	–23.67	52.77	–58.74
(8)	Fatty acid synthesis	FAS	12.14	–13.14	168.42	–190.44	19.23	–20.28	38.78	–43.83
(9)	Beta-oxidation	boxi	–0.45	0.47	–19.09	20.71	–0.64	0.68	–4.41	4.77
(10)	Triglyceride synthesis	TGS	–7.18	7.36	96.77	–112.67	1.07	–1.90	–25.38	26.82
(11)	Lipolysis	Lply	0.37	–0.36	–0.88	0.87	0.13	–0.12	0.71	–0.71

Hepatic TG levels were most sensitive to the reaction rate associated with pyruvate kinase, the rate-limiting enzyme that converts triose phosphate product GA3P to pyruvate for both fructose and glucose. Increasing the enzymatic activity of pyruvate kinase by 10% resulted in 320.09 μmol accumulation of hepatic TG and 74.51 μmol of plasma TG's while decreasing this rate by 10% caused a decline of 357.91 and 83.11 μmol in hepatic and plasma TG's, respectively. Secondly, an increase of 281.15 μmol in hepatic TG and 42.43 μmol in plasma TG levels were observed as the result of increasing the activity rate of KHK by 10%. A decrease of 325.16 μmol in hepatic TG and 49.38 was found when KHK was inhibited by 10%. The reason that the changes in hepatic TGs were greater than that of plasma TGs is because there is around 5% of body lipid stored in the liver under normal conditions, as such the triglyceride level in the liver is ordinarily much higher than that in the plasma. The two significant variations of pyruvate kinase and KHK activity suggests that they are likely to be the key determinants of individual responsiveness to the progression of simple lipid deposition in the liver. However, as KHK is exclusively contributing to the fructose metabolic pathway, it was selected for further investigation in Scenario Three. Apart from PK and KHK, PEPCK, PDC and FAS also expressed with high sensitivity to lipid levels, especially hepatic TG concentrations.

### Scenario Three: Effect on NAFLD Through the Potential Interventional Target KHK

**Figure 5:**
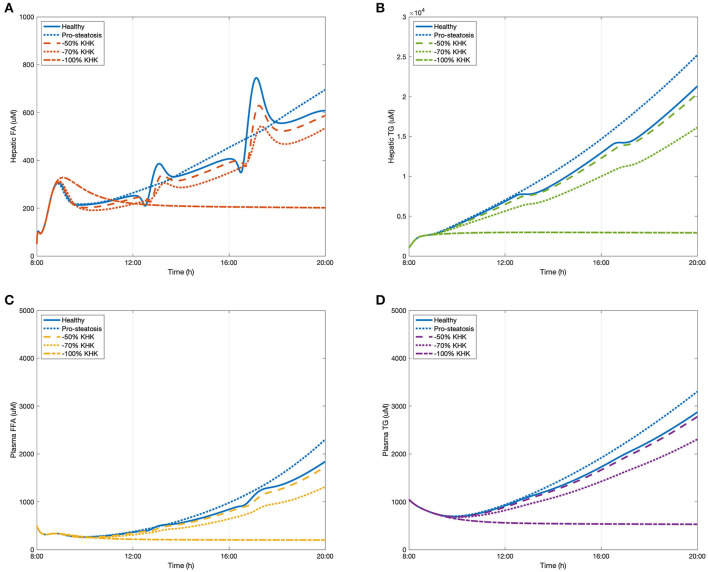
The change of lipid accumulation after inhibiting KHK. **(A)** Hepatic FA, **(B)** hepatic TG, **(C)** plasma FFA, and **(D)** plasma TG.

The early stages of NAFLD are identified by simple lipid accumulation within hepatocytes. Potentially, fructose enables an increase in hepatic *de novo* lipogenesis, thereby contributing to this build up within cells. As demonstrated in Scenario Two, in this process fructokinase (KHK) is of interest as a potential point of intervention. KHK is not limited by adenosine triphosphate (ATP) or citrate availability (as is the case of glucokinase in the glycolytic pathway) as part of the fructose phosphorylation process which potentially delivers a large amount of substrate for lipogenesis. In this scenario, the fructose only diet introduced in Scenario One (100 g/meal) was presented as a representation of a healthy subject. A very high fructose diet (150 g/meal) was employed to simulate the development of a simple pro-steatosis condition, in which a 18.34% increase in hepatic triglycerides was observed. This setting fitted in the criteria of mild steatosis (Petäjä and Yki-Järvinen, 2016). After feeding with the very high fructose diet, three degrees of KHK inhibition were simulated including 50% suppression, 70% suppression, and a full suppression of the enzyme. The results are presented in Figure 5. Inhibiting KHK expression by 50% was able to effectively reduce the hepatic lipid concentrations. Further suppression of KHK by 70% enables lowering of plasma FFA concentration significantly (Figure 5C). When KHK was fully suppressed, hepatic FA, hepatic TG, plasma FFA, and plasma were maintained at a reduced steady-state. These complete inhibitory predictions are consistent with a recent study by Miller et al. (2018) who showed that, after feeding with a high fructose high fat diet, the plasma triglyceride level decreases dramatically in KHK knockout mice when compared to control wild-type animals.

Although we have confidence in the model, we recognize that further validation work is necessary in order to improve and expand the model. The simulations in three scenarios above suggest that the model is robust and it has sufficient detail to present the kinetic relationship between fructose and lipid in the liver. However, the model has its own limitations. Firstly, only acute effects of different carbohydrate diet could be represented by the current model. As the development of NAFLD is chronic and multifactorial, applying only one organ model to investigate the pathological mechanism over a long period may result in cumulative errors. Secondly, the actual dietary intake is more complicated than introduced in this model. The model is designed to give only the response to carbohydrate input. The prediction robustness may be improved if protein and fat consumptions were included for further model expansion. Furthermore, the structure of the liver is complex and the current simulations consider liver as a lumped model that neglects zonation across the liver plate. More comprehensive insight and more accurate information would be provided if the model could be further refined and expanded to include zonated effects.

## Conclusions and Future Work

In this study we introduced a computational model of the hepatic fructose metabolism. The model was validated against experimental data and shown to predict major trends, suggesting that fructose over-consumption leads to dyslipidaemia associated with NAFLD. The model was also used to identify and study a potential regulatory point for novel therapeutic intervention based on the reaction rate sensitivity. As fructokinase is recognized to be the key determinant within the fructose pathway, the effect of fructokinase activity suppression was simulated. When KHK expression was inhibited by 50%, an effective reduction in lipid deposition occurred alleviating simple steatosis while fully inhibited KHK expression induced a notable decrease in lipid accumulation. These results match with experimental data from knock-out animals and should be further corroborated by cellular experimental studies, with the consideration that modification of fructose mediated lipogenesis rather than complete inhibition may be the preferred outcome. In addition, more potential regulatory targets could be tested as candidates for therapeutic treatment.

We believe that organ modeling *in silico* model systems will have numerous applications in developing future therapeutic strategies and represent a future growth area for disease modeling using the quantitative approaches applied by engineers to complex problems. These studies need to involve collaborations between engineers and clinical colleagues.

## Data Availability Statement

All datasets generated for this study are included in the article/Supplementary Material.

## Author Contributions

The computational model was constructed and analyzed by YL under the supervision of ND and IB. YL wrote the manuscript with support from ND and IB. All authors contributed to the article and approved the submitted version.

## Conflict of Interest

The authors declare that the research was conducted in the absence of any commercial or financial relationships that could be construed as a potential conflict of interest.

## Supplementary Material

Table S1 Initial values of variables and parameter values used in the model.

Table S2 Matlab code file of fructose metabolism.
