# Supplementary material for: Computational Modeling of Fructose Metabolism and Development in NAFLD
Source: Front Bioeng Biotechnol. 2020 Jul 22;8:762. doi: 10.3389/fbioe.2020.00762 (PMC7388684; doi:10.3389/fbioe.2020.00762)
Supplement: Table S1 — Initial values of variables and parameter values used in the model. [file Table_1.docx]

Supplementary Material

# Supplementary Table

**Table S1 Initial values of variables and parameter values used in the model**

| Hepatocytes (SH) | Rate Equations | Initial values ($\boldsymbol{u}\boldsymbol{M}$) and references |
| --- | --- | --- |
| Fructose | $\frac{dFru}{dt}=T_{Fru}-\mathbb{R}_{KHK}$ | $\left[ Fru \right]=30;$ [[1](#_ENREF_1)] |
| Fructose-1-Phosphate | $\frac{dF1P}{dt}=\mathbb{R}_{KHK}-\mathbb{R}_{aldB}$ | $\left[ F1P \right]=0.2;$ [[1](#_ENREF_1), [2](#_ENREF_2)] |
| Glucose (Glu) | $\frac{dGlu}{dt}=T_{Glu}-\mathbb{R}_{GK}+\mathbb{R}_{G6Pase}$ | $\left[ Glu \right]=7500$; [[3](#_ENREF_3)] |
| Glucose-6-phosphate (G6P) | $\frac{dG6P}{dt}=\mathbb{R}_{GK}-\mathbb{R}_{G6Pase}+\mathbb{R}_{FBP}-\mathbb{R}_{PFK}$ | $\left[ G6P \right]=102$; [[3](#_ENREF_3)] |
| Dihydroxyacetone phosphate | $\frac{dDHAP}{dt}=\mathbb{R}_{aldB}-\mathbb{R}_{TPI_{DHAP}}+\mathbb{R}_{TPI_{GA3P}}$ | $\left[ DHAP \right]=15;$ [[1](#_ENREF_1)] |
| Glyceraldehyde | $\frac{dGA}{dt}=\mathbb{R}_{aldB}-\mathbb{R}_{Tri}$ | $\left[ GA \right]=1500;$ [[1](#_ENREF_1)] |
| Glyceradehyde-3-phosphate | $\frac{dGA3P}{dt}=\mathbb{R}_{TPI_{DHAP}}-\mathbb{R}_{TPI_{GA3P}}+\mathbb{R}_{Tri}-\mathbb{R}_{PK}+\mathbb{R}_{PEPCK}-\mathbb{R}_{FBP}+\mathbb{R}_{PFK}$ | $\left[ GA3P \right]=480;$ [[1](#_ENREF_1), [3](#_ENREF_3)] |
| Pyruvate/Lactate | $\frac{dPyr}{dt}={T_{Lac}\mathbb{+R}}_{PK}-\mathbb{R}_{PDC}-\mathbb{R}_{PEPCK}$ | $\left[ Pyr \right]=1200;$ [[1](#_ENREF_1), [3](#_ENREF_3)] |
| Acetyl-CoA | $\frac{dACoA}{dt}=\mathbb{R}_{PDC}-{8 \mathbb{R}}_{FAS}+{8 \mathbb{R}}_{boxi}$ | $\left[ ACoA \right]=40;$ [[1](#_ENREF_1), [3](#_ENREF_3)] |
| Fatty Acids (Palmitate) | $\frac{dFA}{dt}={T_{FFA}\mathbb{+R}}_{FAS}-\mathbb{R}_{boxi}-{3 \mathbb{R}}_{TGS}+{3 \mathbb{R}}_{Lply}$ | $\left[ FA \right]=50;$ [[1](#_ENREF_1), [3](#_ENREF_3)] |
| Triglycerides | $\frac{dTG}{dt}={T_{TG}\mathbb{+R}}_{TGS}-\mathbb{R}_{Lply}$ | $\left[ TG \right]=1050;$ [[1](#_ENREF_1), [3](#_ENREF_3)] |
| Hepatic Bloodstream (SHB) | **Rate Equations** | **Initial values (**$\boldsymbol{uM}$**) and references** |
| Fructose | $\frac{d{Fru}_{SHB}}{dt}=-T_{Fru}*R_{HE}+R_{BS}* {(Fru}_{SBC}-{Fru}_{SHB})$ | $\left[ {Fru}_{SHB} \right]=50;$ [[1](#_ENREF_1), [3](#_ENREF_3)] |
| Glucose  (Simplified from [[3](#_ENREF_3)]) | $\frac{d{Glu}_{SHB}}{dt}=-T_{Glu}*R_{HE}+R_{BS}* {(Glu}_{SBC}-{Glu}_{SHB})$ | $\left[ {Glu}_{SHB} \right]=5500;$ [[1](#_ENREF_1), [3](#_ENREF_3)] |
| Pyruvate/Lactate  (Simplified from [[3](#_ENREF_3)]) | $\frac{d{Pyr}_{SHB}}{dt}=-T_{Pyr}*R_{HE}+R_{BS}* {(Pyr}_{SBC}-{Pyr}_{SHB})$ | $\left[ {Pyr}_{SHB} \right]=1000;$ [[1](#_ENREF_1), [3](#_ENREF_3)] |
| Fatty acids (Palmitate)  (Simplified from [[3](#_ENREF_3)]) | $\frac{d{FA}_{SHB}}{dt}=-T_{FA}*R_{HE}+R_{BS}* {(FA}_{SBC}-{FA}_{SHB})$ | $\left[ {FA}_{SHB} \right]=500;$ [[1](#_ENREF_1), [3](#_ENREF_3)] |
| Triglyceride  (Simplified from [[3](#_ENREF_3)]) | $\frac{d{TG}_{SHB}}{dt}=-T_{TG}*R_{HE}+R_{BS}* {(TG}_{SBC}-{TG}_{SHB})$ | $\left[ {TG}_{SHB} \right]=1050;$ [[1](#_ENREF_1), [3](#_ENREF_3)] |
| Section Bloodstream Circulation (SBC) | **Rate Equations** | **Initial values (**$\boldsymbol{uM}$**) and references** |
| Fructose | $\frac{d{Fru}_{SBC}}{dt}={Meal}_{Fru}+C_{Fru}$ | $\left[ {Fru}_{SBC} \right]=50;$ [[1](#_ENREF_1), [3](#_ENREF_3)] |
| Glucose  (Simplified from [[3](#_ENREF_3)]) | $\frac{d{Glu}_{SBC}}{dt}={Meal}_{Glu}+C_{Glu}-{USE}_{Glu}-{UP}_{FA}-{UP}_{TG}$ | $\left[ {Glu}_{SBC} \right]=5500;$ [[1](#_ENREF_1), [3](#_ENREF_3)] |
| Pyruvate/Lactate  (Simplified from [[3](#_ENREF_3)]) | $\frac{d{Pyr}_{SBC}}{dt}=C_{Pyr}$ | $\left[ {Pyr}_{SBC} \right]=1000;$ [[1](#_ENREF_1), [3](#_ENREF_3)] |
| Fatty acids (Palmitate)  (Simplified from [[3](#_ENREF_3)]) | $\frac{d{FA}_{SBC}}{dt}=C_{FA}-{USE}_{FA}+{UP}_{FA}$ | $\left[ {FA}_{SBC} \right]=500;$ [[1](#_ENREF_1), [3](#_ENREF_3)] |
| Triglyceride  (Simplified from [[3](#_ENREF_3)]) | $\frac{d{TG}_{SBC}}{dt}=C_{TG}{-USE}_{TG}+{UP}_{TG}$ | $\left[ {TG}_{SBC} \right]=1050;$ [[1](#_ENREF_1), [3](#_ENREF_3)] |
| Hepatic Enzymes /Reactions in SH | **Rate Functions** | **Parameter values and references (Km unit: μM; Reaction rate unit: μM/s)** |
| Fructokinase | $\mathbb{R}_{KHK}=V_{KHK}*\frac{{Fru}^{nFru}}{{{Km}_{KHK}}^{nFru}+{Fru}^{nFru}}*\frac{{ATP}^{nATP}}{{{Km}_{ATP}}^{nATP}+{ATP}^{nATP}}$ | ${Km}_{KHK}=800;$ [[4](#_ENREF_4)]  ${Km}_{ATP}=1430;$ [[5](#_ENREF_5)]  $V_{KHK}=4.5;$ [[6](#_ENREF_6)] |
| Aldolase B | $\mathbb{R}_{aldB}=V_{aldB}*\frac{{F1P}^{nF1P}}{{{Km}_{F1P}}^{nF1P}+{F1P}^{nF1P}}$ | ${Km}_{F1P}=230;$ [[7](#_ENREF_7)]  $V_{aldB}=1.7;$ [[8](#_ENREF_8)] |
| Triose phosphate isomerase | $\mathbb{R}_{TPI\_DHAP}=V_{TPI\_DHAP}*\frac{{DHAP}^{nDHAP}}{{{Km}_{DHAP}}^{nDHAP}+{DHAP}^{nDHAP}}$  $\mathbb{R}_{TPI\_GA3P}=V_{TPI\_GA3P}*\frac{{GA3P}^{nGA3P}}{{{Km}_{TPIGA3P}}^{nGA3P}+{GA3P}^{nGA3P}}$ | ${Km}_{DHAP}=590;$ [[9-11](#_ENREF_9)]  ${Km}_{TPIGA3P}=400;$ [[11](#_ENREF_11)]  $V_{TPI\_DHAP}=2.7;$ [[9-11](#_ENREF_9)]  $V_{TPI\_GA3P}=0.05;$ [[9-11](#_ENREF_9)] |
| Triokinase | $\mathbb{R}_{Tri}=V_{Tri}*\frac{{GA}^{nGA}}{{{Km}_{GA}}^{nGA}+{GA}^{nGA}}*\frac{{ATP_{{Mg}^{2-}}}^{nATP_{{Mg}^{2-}}}}{{{Km}_{ATP_{{Mg}^{2-}}}}^{nATP_{{Mg}^{2-}}}+{ATP_{{Mg}^{2-}}}^{nATP_{{Mg}^{2-}}}}*\left( 1-\beta_{ATP}\frac{ATP}{K_{i}^{ATP}+ATP} \right)\left( 1-\beta_{ADP}\frac{ADP}{K_{i}^{ADP}+ADP} \right)$ | ${Km}_{GA}=18;$ [[12](#_ENREF_12)]  ${Km}_{ATP_{{Mg}^{2-}}}=770;$ [[13](#_ENREF_13)]  $K_{i}^{ATP}=380;$ [[13](#_ENREF_13)]  $K_{i}^{ADP}=1100;$ [[13](#_ENREF_13)]  $V_{Tri}=16.7;$ [[12](#_ENREF_12)] |
| Pyruvate kinase | $\mathbb{R}_{PK}=V_{PK}*\frac{{GA3P}^{nGA3P}}{{{Km}_{GA3P}}^{nGA3P}+{GA3P}^{nGA3P}}*\frac{{ADP}^{nADPpk}}{{{Km}_{ADPpk}}^{nADPpk}+{ADP}^{nADPpk}}*\left( 1-\beta_{ACoA-PK}\frac{ACoA}{K_{i}^{ACoA-PK}+ACoA} \right)$ | ${Km}_{GA3P}=250;$ [[14-16](#_ENREF_14)]  ${Km}_{ADPpk}=240;$ [[17](#_ENREF_17)]  $K_{i}^{ACoA-PK}=30;$ [[18](#_ENREF_18)]  $\beta_{ACoA-PK}=0.8;$ [[18](#_ENREF_18)]  $V_{PK}=87;$ [[3](#_ENREF_3)] |
| Phosphoenolpyruvate carboxykinase | $\mathbb{R}_{PEPCK}=V_{PEPCK}*\frac{Pyr}{K_{m}^{PEPCK}+Pyr}*\frac{ATP}{K_{m}^{ATPpepck}+ATP}*\frac{GTP}{K_{m}^{GTP}+GTP}$ | $K_{m}^{PEPCK}=500;$ [[14-16](#_ENREF_14)]  $K_{m}^{ATPpepck}=10;$ [[3](#_ENREF_3)]  $K_{m}^{GTP}=64;$ [[19](#_ENREF_19)]  $V_{PEPCK}=35;$ [[3](#_ENREF_3)] |
| Pyruvate oxidation | $\mathbb{R}_{PDC}=V_{PDC}*\frac{Pyr}{K_{m}^{Pyr}+Pyr}*\left( 1-\beta_{ACoA-PDC}\frac{ACoA}{ACoA+k_{i}^{CoA-pyr}} \right)$ | $K_{m}^{Pyr}=540;$ [[14-16](#_ENREF_14), [20](#_ENREF_20)]  $k_{i}^{CoA-pyr}=35;$ [[21](#_ENREF_21)]  $\beta_{ACoA-PDC}=1;$ [[21](#_ENREF_21)]  $V_{PDC}=15;$ [[3](#_ENREF_3)] |
| Fatty acid synthesis | $\mathbb{R}_{FAS}{=V}_{FAS}*\frac{ACoA}{K_{m}^{ACoA}+ACoA}*\frac{ATP}{K_{m}^{ATPfas}+ATP}*\left( 1-\beta_{FA}\frac{FA}{FA+k_{i}^{FA-inhib}} \right)$ | $K_{m}^{ACoA}=58;$ [[22](#_ENREF_22)]  $K_{m}^{ATPfas}=120;$ [[22](#_ENREF_22)]  $\beta_{FA}=1;$ [[23](#_ENREF_23)]  $k_{i}^{FA-inhib}=300;$ [[23](#_ENREF_23)]  $V_{FAS}=4;$ [[3](#_ENREF_3)] |
| Beta-oxidation | $\mathbb{R}_{boxi}{=V}_{boxi}*\frac{FA}{K_{m}^{boxi}+FA}*\frac{ATP}{K_{m}^{ATPboxi}+ATP}*\left( 1-\beta_{boxi}\frac{ACoA}{ACoA+k_{i}^{CoA-boxi}} \right)*\left( 1-\beta_{PPAR\alpha}\frac{F1P}{F1P+k_{i}^{F1P-inhib}} \right)$ | $K_{m}^{boxi}=5;$ [[24-26](#_ENREF_24)]  $K_{m}^{ATPboxi}=87;$ [[27](#_ENREF_27)]  $k_{i}^{CoA-boxi}=47.8;$ [[28](#_ENREF_28)]  $k_{i}^{F1P-inhib}=100;$ [[29](#_ENREF_29)]  $\beta_{boxi}=0.4;$ [[28](#_ENREF_28)]  $\beta_{PPAR\alpha}=1;$ [[29](#_ENREF_29)]  $V_{boxi}=3.3;$ [[3](#_ENREF_3)] |
| Triglyceride synthesis | ${\mathbb{R}_{TGS}=V}_{TGS}*\frac{FA}{K_{m}^{FA}+FA}*\frac{GA3P}{K_{m}^{TGSGA3P}+GA3P}$ | $K_{m}^{FA}=645;$ [[24-26](#_ENREF_24)]  $K_{m}^{TGSGA3P}=460;$ [[30](#_ENREF_30)]  $V_{TGS}=10;$ [[3](#_ENREF_3)] |
| Lipolysis | $\mathbb{R}_{Lply}=V_{Lply}*\frac{TG}{K_{m}^{TG}+TG}$ | $K_{m}^{TG}=50715;$ [[31](#_ENREF_31), [32](#_ENREF_32)]  $V_{Lply}=0.085;$ [[3](#_ENREF_3)] |
| Transport Variables in SHB | **Rate Functions for cross-membrane transportation** | **Parameter values and references (Km unit: μM; Reaction rate unit: μM/s)** |
| Fructose | $T_{Fru}=V_{GLUT2}^{pump}*\frac{{Fru}_{SHB}}{K_{m}^{GLUT2-pump}+{Fru}_{SHB}}+V_{GLUT2}^{ex}*\frac{{Fru}_{SHB}-{Fru}_{SH}}{K_{m}^{GLUT2-ex}+{Fru}_{SHB}+{Fru}_{SH}}+$  $V_{GLUT5}^{pump}*\frac{{Fru}_{SHB}}{K_{m}^{GLUT5-pump}+{Fru}_{SHB}}+V_{GLUT5}^{ex}*\frac{{Fru}_{SHB}-{Fru}_{SH}}{K_{m}^{GLUT5-ex}+{Fru}_{SHB}+{Fru}_{SH}}$ | $K_{M}^{GLUT2-pump}=76000;$ [[33](#_ENREF_33)]  $K_{m}^{GLUT2-ex}=76000;$ [[33](#_ENREF_33)]  $K_{m}^{GLUT5-pump}=6000;$ [[33-35](#_ENREF_33)]  $K_{m}^{GLUT5-ex}=6000;$ [[33-35](#_ENREF_33)]  $V_{GLUT2}^{pump}=10;$ [[33](#_ENREF_33)]  $V_{GLUT2}^{ex}=30;$ [[33](#_ENREF_33)]  $V_{GLUT5}^{pump}=20;$ [[33-35](#_ENREF_33)]  $V_{GLUT5}^{ex}=60;$ [[33-35](#_ENREF_33)] |
| Glucose | $T_{Glu}=V_{GLUTG}^{pump}*\frac{{Glu}_{SHB}}{K_{m}^{GLUTG-pump}+{Glu}_{SHB}}+V_{GLUTG}^{ex}*\frac{{Glu}_{SHB}-{Glu}_{SH}}{K_{m}^{GLUTG-ex}+{Glu}_{SHB}+{Glu}_{SH}}$ | $K_{m}^{GLUTG-pump}=17000;$ [[33](#_ENREF_33), [36](#_ENREF_36), [37](#_ENREF_37)]  $K_{m}^{GLUTG-ex}=17000;$ [[33](#_ENREF_33), [36](#_ENREF_36), [37](#_ENREF_37)]  $V_{GLUTG}^{pump}=118;$ [[3](#_ENREF_3)]  $V_{GLUTG}^{ex}=224;$ [[3](#_ENREF_3)] |
| Pyruvate/Lactate | $T_{Pyr}=V_{Pyr}^{ex}*\frac{{Pyr}_{SHB}-{Pyr}_{SH}}{K_{m}^{Pyr-ex}+{Pyr}_{SHB}+{Pyr}_{SH}}$ | $K_{m}^{Pyr-ex}=$1200; [[3](#_ENREF_3)]  $V_{Pyr}^{ex}=200;$ [[3](#_ENREF_3)] |
| Fatty acids (Palmitate) | $T_{FA}=V_{FA}^{ex}*\frac{{FA}_{SHB}-{FA}_{SH}}{K_{m}^{FA-ex}+{FA}_{SHB}+{FFA}_{SH}}+V_{active}* \frac{FA_{SHB}}{\left( K_{m}^{active}+FA_{SHB} \right)}\left( 1+\frac{Ins}{{Ins}_{ref}^{active}} \right)$ | $K_{m}^{FA-ex}=200;$ [[3](#_ENREF_3)]  $K_{m}^{active}=2;$ [[3](#_ENREF_3)]  $V_{FA}^{ex}=1.45;$ [[3](#_ENREF_3)]  $V_{active}=0.08;$ [[3](#_ENREF_3)] |
| Triglyceride | $T_{TG}=V_{TG}^{ex}*\frac{\left( {TG}_{SHB}-\frac{{TG}_{SH}}{{TG}_{ref}} \right)}{K_{m}^{TG-ex}+{TG}_{SHB}+\frac{{TG}_{SH}}{{TG}_{ref}}}- V_{out}*\frac{{TG}_{SH}}{\left( K_{m}^{out}+{TG}_{SH} \right)}$ | $K_{m}^{TG-ex}=1000;$ [[3](#_ENREF_3)]  $K_{m}^{out}=33810;$ [[3](#_ENREF_3)]  $V_{TG}^{ex}=0.6;$ [[3](#_ENREF_3)]  $V_{out}=0.3;$ [[3](#_ENREF_3)] |
| Glucose input variables  (Simplified from [[3](#_ENREF_3)]) | **Rate Functions** | **Parameter values and references (Km unit: μM; Reaction rate unit: μM/s)** |
| Glucokinase | $\mathbb{R}_{GK}=V_{GK}*\frac{{Glu}^{nGlu}}{{{Km}_{Glu}}^{nGlu}+{Glu}^{nGlu}}*\frac{{ATP}^{nATP}}{{{Km}_{ATPgk}}^{nATP}+{ATP}^{nATP}}*(1-\frac{G6P}{G6P+k_{i}^{G6P}})$ | ${Km}_{Glu}=7500;$ [[3](#_ENREF_3)]  ${Km}_{ATPgk}=240;$ [[3](#_ENREF_3)]  $k_{i}^{G6P}=240;$ [[3](#_ENREF_3)]  $V_{GK}=$132.16; [[3](#_ENREF_3)] |
| Glucose-6-phosphatase | $\mathbb{R}_{G6Pase}=V_{G6Pase}*\frac{G6P}{K_{m}^{G6Pase}+G6P}$ | $K_{m}^{G6Pase}=2410;$ [[3](#_ENREF_3)]  $V_{G6Pase}=370;$ [[3](#_ENREF_3)] |
| Fructose-bisphosphatase | $\mathbb{R}_{FBP}=V_{FBP}*\frac{GA3P}{K_{m}^{FBP}+GA3P}$ | $K_{m}^{FBP}=250;$ [[3](#_ENREF_3)]  $V_{FBP}=68;$ [[3](#_ENREF_3)] |
| Phosphofructokinase | $\mathbb{R}_{PFK}=V_{PFK}*\frac{G6P}{K_{m}^{PFK}+G6P}*\frac{ATP}{{Km}_{ATPpfk}+ATP}*\left( 1-\frac{ATP}{ATP+k_{i}^{ATPfpk}}*\frac{ADP}{ADP+k_{i}^{ADPfpk}} \right)*(1-\beta_{PFK}\frac{GA3P}{GA3P+k_{i}^{GA3Ppfk}})$ | $K_{m}^{PFK}=5;$ [[3](#_ENREF_3)]  ${Km}_{ATPpfk}=42.5;$ [[3](#_ENREF_3)]  $k_{i}^{ATPfpk}=2100;$ [[3](#_ENREF_3)]  $k_{i}^{ADPfpk}=83.6;$ [[3](#_ENREF_3)]  $k_{i}^{GA3Ppfk}=20.7;$ [[3](#_ENREF_3)]  $\beta_{PFK}=0.75;$ [[3](#_ENREF_3)]  $V_{PFK}=160;$ [[3](#_ENREF_3)] |

# References

1. Sengupta, A., et al., *HEPNet: A Knowledge Base Model of Human Energy Pool Network for Predicting the Energy Availability Status of an Individual.* PloS one, 2015. **10**(6): p. e0127918.

2. Rodrigues, J.R., et al., *Bifunctional Homodimeric Triokinase/FMN Cyclase CONTRIBUTION OF PROTEIN DOMAINS TO THE ACTIVITIES OF THE HUMAN ENZYME AND MOLECULAR DYNAMICS SIMULATION OF DOMAIN MOVEMENTS.* Journal of Biological Chemistry, 2014. **289**(15): p. 10620-10636.

3. Ashworth, W.B., N.A. Davies, and I.D.L. Bogle, *A computational model of hepatic energy metabolism: understanding zonated damage and steatosis in NAFLD.* PLoS Comput Biol, 2016. **12**(9): p. e1005105.

4. Bais, R., et al., *The purification and properties of human liver ketohexokinase. A role for ketohexokinase and fructose-bisphosphate aldolase in the metabolic production of oxalate from xylitol.* Biochemical journal, 1985. **230**(1): p. 53-60.

5. Phillips, M.I. and D.R. Davies, *The mechanism of guanosine triphosphate depletion in the liver after a fructose load. The role of fructokinase.* Biochemical Journal, 1985. **228**(3): p. 667-671.

6. Asipu, A., et al., *Properties of normal and mutant recombinant human ketohexokinases and implications for the pathogenesis of essential fructosuria.* Diabetes, 2003. **52**(9): p. 2426-32.

7. Doyle, S.A. and D.R. Tolan, *Characterization of recombinant human aldolase B and purification by metal chelate chromatography.* Biochem Biophys Res Commun, 1995. **206**(3): p. 902-8.

8. Dawson, N.J., K.K. Biggar, and K.B. Storey, *Characterization of fructose-1, 6-bisphosphate aldolase during anoxia in the tolerant turtle, Trachemys scripta elegans: an assessment of enzyme activity, expression and structure.* PloS one, 2013. **8**(7): p. e68830.

9. Yuan, P., et al., *Isolation and characterization of triosephosphate isomerase isozymes from human placenta.* Archives of biochemistry and biophysics, 1979. **198**(1): p. 42-52.

10. Gracy, R.W., *[94] Triosephosphate isomerase from human erythrocytes.* Methods in enzymology, 1975. **41**: p. 442-447.

11. Snyder, R. and E.W. Lee, *[91] Triosephosphate isomerase from human and horse liver.* Methods in enzymology, 1975. **41**: p. 430-434.

12. Sillero, M., A. Sillero, and A. Sols, *Enzymes involved in fructose metabolism in liver and the glyceraldehyde metabolic crossroads.* European Journal of Biochemistry, 1969. **10**(2): p. 345-350.

13. Frandsen, E.K. and N. Grunnet, *Kinetic properties of triokinase from rat liver.* European Journal of Biochemistry, 1971. **23**(3): p. 588-592.

14. Daly, M.E., et al., *Acute effects on insulin sensitivity and diurnal metabolic profiles of a high-sucrose compared with a high-starch diet.* The American journal of clinical nutrition, 1998. **67**(6): p. 1186-1196.

15. Albe, K.R., M.H. Butler, and B.E. Wright, *Cellular concentrations of enzymes and their substrates.* Journal of theoretical biology, 1990. **143**(2): p. 163-195.

16. Taylor, R., et al., *Direct assessment of liver glycogen storage by 13C nuclear magnetic resonance spectroscopy and regulation of glucose homeostasis after a mixed meal in normal subjects.* The Journal of clinical investigation, 1996. **97**(1): p. 126-132.

17. Dombrauckas, J.D., B.D. Santarsiero, and A.D. Mesecar, *Structural basis for tumor pyruvate kinase M2 allosteric regulation and catalysis.* Biochemistry, 2005. **44**(27): p. 9417-9429.

18. Weber, G., M.A. Lea, and N.B. Stamm, *Inhibition of pyruvate kinase and glucokinase by acetyl CoA and inhibition of glucokinase by phosphoenolpyruvate.* Life Sciences, 1967. **6**(22): p. 2441-2452.

19. Dharmarajan, L., et al., *Tyr235 of human cytosolic phosphoenolpyruvate carboxykinase influences catalysis through an anion–quadrupole interaction with phosphoenolpyruvate carboxylate.* The FEBS journal, 2008. **275**(23): p. 5810-5819.

20. Ainscow, E.K. and M.D. Brand, *Top‐down control analysis of ATP turnover, glycolysis and oxidative phosphorylation in rat hepatocytes.* European Journal of Biochemistry, 1999. **263**(3): p. 671-685.

21. Kiselevsky, Y.V., S.A. Ostrovtsova, and S.A. Strumilo, *Kinetic characterization of the pyruvate and oxoglutarate dehydrogenase complexes from human heart.* Acta Biochimica Polonica, 1990. **37**(1): p. 135-139.

22. Cheng, D., et al., *Expression, purification, and characterization of human and rat acetyl cfenzyme A carboxylase (ACC) isozymes.* Protein expression and purification, 2007. **51**(1): p. 11-21.

23. Reaven, G.M., et al., *Measurement of plasma glucose, free fatty acid, lactate, and insulin for 24 h in patients with NIDDM.* Diabetes, 1988. **37**(8): p. 1020-1024.

24. Kim, J.-H., T.M. Lewin, and R.A. Coleman, *Expression and Characterization of Recombinant Rat Acyl-CoA Synthetases 1, 4, and 5 Selective Inhibition By Triacsin C And Thiazolidinediones.* Journal of Biological Chemistry, 2001. **276**(27): p. 24667-24673.

25. Van Horn, C.G., et al., *Characterization of recombinant long-chain rat acyl-CoA synthetase isoforms 3 and 6: identification of a novel variant of isoform 6.* Biochemistry, 2005. **44**(5): p. 1635-1642.

26. Marcel, Y.L. and G. Suzue, *Kinetic studies on the specificity of long chain acyl coenzyme A synthetase from rat liver microsomes.* Journal of Biological Chemistry, 1972. **247**(14): p. 4433-4436.

27. Stinnett, L., T.M. Lewin, and R.A. Coleman, *Mutagenesis of rat acyl-CoA synthetase 4 indicates amino acids that contribute to fatty acid binding.* Biochimica et Biophysica Acta (BBA)-Molecular and Cell Biology of Lipids, 2007. **1771**(1): p. 119-125.

28. Zierz, S. and A.G. Engel, *Different sites of inhibition of carnitine palmitoyltransferase by malonyl-CoA, and by acetyl-CoA and CoA, in human skeletal muscle.* Biochemical Journal, 1987. **245**(1): p. 205-209.

29. Nomura, K. and T. Yamanouchi, *The role of fructose-enriched diets in mechanisms of nonalcoholic fatty liver disease.* The Journal of nutritional biochemistry, 2012. **23**(3): p. 203-208.

30. Vancura, A. and D. Haldar, *Purification and characterization of glycerophosphate acyltransferase from rat liver mitochondria.* Journal of Biological Chemistry, 1994. **269**(44): p. 27209-27215.

31. KAPLAN, A. and M.-H. Teng, *Interaction of beef liver lipase with mixed micelles of tripalmitin and Triton X-100.* Journal of Lipid Research, 1971. **12**(3): p. 324-330.

32. Chakraborty, K. and R.P. Raj, *An extra-cellular alkaline metallolipase from Bacillus licheniformisMTCC 6824: purification and biochemical characterization.* Food chemistry, 2008. **109**(4): p. 727-736.

33. Zhao, F.-Q. and A.F. Keating, *Functional properties and genomics of glucose transporters.* Current genomics, 2007. **8**(2): p. 113-128.

34. Burant, C., et al., *Fructose transporter in human spermatozoa and small intestine is GLUT5.* Journal of Biological Chemistry, 1992. **267**(21): p. 14523-14526.

35. Karim, S., D.H. Adams, and P.F. Lalor, *Hepatic expression and cellular distribution of the glucose transporter family.* World journal of gastroenterology: WJG, 2012. **18**(46): p. 6771.

36. Thorens, B., *Molecular and Cellular Physiology of GLUT-2, a High-K~ m Facilitated Diffusion Glucose Transporter.* International review of cytology, 1992: p. 209-209.

37. Laughlin, M., *Normal roles for dietary fructose in carbohydrate metabolism.* Nutrients, 2014. **6**(8): p. 3117-3129.
