# Supplementary material for: Computational Modeling of Fructose Metabolism and Development in NAFLD
Source: Front Bioeng Biotechnol. 2020 Jul 22;8:762. doi: 10.3389/fbioe.2020.00762 (PMC7388684; doi:10.3389/fbioe.2020.00762)
Supplement: Table S2 — Matlab code file of fructose metabolism. [file Table_2.pdf]

```
function dydt=Fructose_YL(t,y)

% 1. Function Inputs

% 1.1 Plasma section
B_F = y(1); % Blood Fructose
B_G = y(2); % Blood Glucose
B_L = y(3); % Blood Lactate
B_FFA = y(4); % Blood Free Fatty Acids
B_TG = y(5); % Blood Triglyceride
P_Ins = y(6); % Pancreas Insulin
P_Gcg = y(7);  % Pancreas Glucagon

% 1.2 Bloodstream Exchange section --- Hepatic blood
EX_F = y(8); % Fructose Exchange
EX_G = y(9); % Glucose Exchange
EX_L = y(10); % Lactate Exchange
EX_FFA = y(11); % Free Fatty Acids Exchange
EX_TG = y(12); % Triglyceride Exchange
EX_Ins = y(13); % Insulin Exchange
EX_Gcg = y(14);  % Glucagon Exchange

% 1.3 Hepatic section

Fru = y(15); % Hepatic fructose
F1P = y(16); % Hepatic Fructose-1-phosphate
Glu = y(17); % Hepatic Glucose
G6P = y(18); % Hepatic Glucose-6-phosphate
DHAP = y(19); % Hepatic Dihydroxyacetone phosphate
GA = y(20); % Hepatic Glyceraldehyde
GA3P = y(21); % Hepatic D-glyceraldehyde 3-phosphate
pyr = y(22); % Hepatic Pyruvate/Lactate
acoa = y(23); % Hepatic Acetyl-CoA
FA = y(24); % Hepatic Fatty acid
TG = y(25); % Hepatic Triglyceride

% 1.4 Conditional control
lipoi = y(26);
lipol = y(27);
trii = y(28);
tril = y(29);

% 2. Input of fructose and glucose in the liver
% Input of fructose (uptake rate based on portal vein concentration)
DriveFru = 4.5;
DriveGlu = 12.34;
timeref = 28800;
pow = 6;
```

```
% 3. Parameter values:
% 3.1 Bloodstream dynamic
R_BS = 0.167;
R_RL = 5.25;
R_HE = 4;

% 3.2 Transportation
% 3.2.1 Fructose
v_glut2 = 10;
k_glut2 = 76000;
v_ex2 = 30;
k_ex2 = 76000;
v_glut5 = 20;
k_glut5 = 6000;
v_ex5 = 60;
k_ex5 = 6000;
% 3.2.2 Glucose
v_glutG = 118;
k_glutG = 17000;
v_exG = 224;
k_exG = 17000;
% 3.2.3 Pyruvate/Lactate
v_exLac = 200;
k_exLac = 1200;
% 3.2.5 Free fatty acids
v_exFA = 1.45;
k_exFA = 200;
v_act = 0.08;
InsRef = 21.33;
k_act = 2;
% 3.2.6 Triglycerides
v_outTG = 0.3;
k_outTG = 33810;
v_exTG=0.6;
k_exTG=1000;
TTGRef=6.5;

% 3.3 [Out]Adipose & Muscle (rest of the body usage)
% 3.3.1 Glucose uptake by the rest of the body
k_useG = 1000;
v_useG = 5.15;
% 3.3.2 FFA uptake by the rest of the body
k_gcgFFA=1;
k_insFFA=0.6;
v_useFFA=1.4;
k_useFFA=100;
% 3.3.3 Fructose uptake
v_useF = 5.15;
k_useF = 1000;
% 3.3.3 DNL in the rest of the body
```

```
k_useDNL=4500;
v_useDNL=0.11;
% 3.3.4 TG Synthesis in the rest of the body
v_useTGS = 3;
k_useTGSg=10000;
k_useTGSffa=645;
% 3.3.5 TG consumption by the rest of the body
v_useLil=2.3;
k_useLil=2000;

% 3.4 Michaelis constant & enzymatic maximum rates (Vmax)
% Unit concentration(uM), time(s), rate (uM/s)

% Hepatic section
k_fru = 800;
v_khk = 4.5;
k_ATPkhk = 1430;
k_F1P = 230;
v_ald = 1.7;
k_DHAP = 590;
v_TPI_DHAP = 2.7;
k_GA3P_TPI= 400;
v_TPI_GA3P = 0.05;
k_GA = 18;
v_Tri = 16.7;
k_ATPMg = 770;
kiATPiTri = 380;
kiADPiTri = 1100;
k_GA3P_PK =250;
v_PK = 87;
k_ADPpk = 240;
eff = 0.8;
kiacoaPK = 30;
k_pyr = 540;
v_PDC = 15;
kiacoaPDC = 35;
v_PEPCK = 35;
k_PEPCK = 500;
k_ATPglu = 10;
k_GTPglu = 64;
% Lipogenesis
v_lipg = 4;
k_acoa = 58;
k_ATPlipg = 120;
kiFA = 300;
% Beta-oxidation
v_boxi = 3.3;
k_boxi = 5;
effac = 1;
kiacbox = 47.8;
```

```
k_ATPbox = 87;
effPPAR = 1;
kiF1P = 100;
% Triglyceride Synthesis
v_tsyn = 10;
k_GA3P_tsyn = 460;
k_FA = 645;
% Lipolysis
v_lipo = 0.085;
k_TG = 50715;
% Glucose combination (simplified from Ashworth's model)
v_GK = 132.16;
nglu = 1.4;
k_glu = 7500;
k_ATPgk = 240;
kig6pGK = 240;
nig6pGK = 4;
v_G6P = 370;
k_G6P = 2410;
hFBPPFKref = 10;
v_FBP = 68;
k_FBP = 250;
v_PFK = 160;
k_PFK = 5;
k_ATPpfk = 42.5;
kiatpPFK = 2100;
kiadpPFK = 83.6;
effpfk = 0.75;
kiga3p = 20.7;
hPEPCKref = 8.5;
hPKref = 4;
hACOAInsref = 5;
hACOAGcgref = 3;
hDNLInsref = 3;
hDNLGcgref = 7;
hboxiInsref = 2.5;
hboxiGcgref = 7;
hTGSInsref = 4;
hTGSGcgref = 4;
hLplyInsref = 4;
hplyGcgref = 5;
ATP = 2780;
ADP = 285;
GTP = 284;

% Pancreatic Hormone Release and Transport in Blood
% Insulin and Glucagon setting based on Heatherington's model
rel=5;
gr=3;
gri=2;
```

```
tauL=0.036;
tLg=1.3;
nL=2;
ML=3;
tauI=0.075;
tIg=0.9;
nI=2;
MI=4;
BloodScale=4.9999995e-4;
mins=60;
P_Ins = 266.67*P_Ins;
P_Gcg = 125*P_Gcg;
EX_Ins = 266.67*EX_Ins;
EX_Gcg = 125*EX_Gcg;
slow=15;
fast=0.07;
Lreftriw=0.3;
Ireftriw=3;
tti=1000;
ttl=10000;
tli=1000;
tll=10000;
LrefLipow=0.3;
Ireflipow=3;

% 4. Dietary Inputs
% 4.1 Dietary Input of Fructose
if t<=43200
   M_Fru = (DriveFru*((sin(2*pi*t/(timeref))).^pow));
elseif (129600>=t) && (t>=86400)
   M_Fru = (DriveFru*((sin(2*pi*t/(timeref))).^pow));
else
   M_Fru = 0;
end


% 4.2 Dietary Input of Glucose
if t<=43200
   M_Glu = (DriveGlu*((sin(2*pi*t/(timeref))).^pow));
elseif (129600>=t) && (t>=86400)
   M_Glu = (DriveGlu*((sin(2*pi*t/(timeref))).^pow));
else
   M_Glu = 0;
end



% 5. [In]Cross-membrane transportation
% 5.1 Fructose transportation
T_GLUT2 = v_glut2*EX_F/(k_glut2+EX_F) + v_ex2*(EX_F-Fru)/(k_ex2+EX_F+Fru);
```

```
T_GLUT5 = v_glut5*EX_F/(k_glut5+EX_F) + v_ex5*(EX_F-Fru)/(k_ex5+EX_F+Fru);
% 5.2 Glucose transportation
T_GLUTG = v_glutG*EX_G/(k_glutG+EX_G) + v_exG*(EX_G-Glu)/
(k_exG+EX_G+Glu);
% 5.3 Lactate transportation
T_Lac=v_exLac*(EX_L-pyr)/(k_exLac+EX_L+pyr);
% 5.4 Free fatty acids transportation
T_FA = v_exFA*(EX_FFA-FA)/(k_exFA+EX_FFA+FA)+v_act*EX_FFA*(1+EX_Ins/
InsRef)/(EX_FFA+k_act);
% Triglyceride transportation
T_TG =v_exTG*(EX_TG-TG/TTGRef)/(k_exTG+TG/TTGRef+EX_TG)-
(v_outTG*TG/(k_outTG+TG));
% 6. [Out]Adipose & Muscle (rest of the body usage)
% Glucose usage
U_G=v_useG*B_G/(k_useG+B_G);
% FFA usage
U_FFA=v_useFFA*B_FFA/(B_FFA+k_useFFA)*((P_Ins+k_insFFA)/
(P_Gcg+k_gcgFFA));
% DNL
irefdenog=7;
lrefdenog=2;
U_DNL = v_useDNL*B_G/(B_G+k_useDNL)*turn((1+P_Ins/irefdenog-P_Gcg/
lrefdenog));
% Glucose and FFA removal (Adipose & Muscle Triglyceride Synthesis)
if (P_Ins/Ireftriw-trii)>0
   dtriidt=(P_Ins/Ireftriw-trii)/tti;
else
   dtriidt=(P_Ins/Ireftriw-trii)/(slow*tti);
end

if (P_Gcg/Lreftriw-tril)>0
   dtrildt=(P_Gcg/Lreftriw-tril)/ttl;
else
   dtrildt=(P_Gcg/Lreftriw-tril)/(fast*ttl);
end

U_TGS=v_useTGS*(B_G*B_FFA)/
((k_useTGSg+B_G)*(k_useTGSffa+B_FFA))*turn(1+trii-tril);

% Triglyceride Removal (Adipose & Muscle lipolysis)

if (P_Ins/Ireflipow-lipoi)>0
   dlipoidt=(P_Ins/Ireflipow-lipoi)/tli;
else
   dlipoidt=(P_Ins/Ireflipow-lipoi)/(slow*tli);
end


if (P_Gcg/LrefLipow-lipol)>0
   dlipoldt=(P_Gcg/LrefLipow-lipol)/tll;
```

```
else
   dlipoldt=(P_Gcg/LrefLipow-lipol)/(fast*tll);
end
U_LiI = v_useLiI*B_TG/(B_TG+k_useLiI)*turn(1-lipoi+lipol);


% 7. Hepatic reaction rate of each enzyme
% Fructose
r_khk = v_khk*1/(k_fru/Fru+1);
r_ATPkhk = 1/(k_ATPkhk/ATP+1);
r_ald = v_ald*1/(k_F1P/F1P+1);
r_TPI_DHAP = v_TPI_DHAP*1/(k_DHAP/DHAP+1);
r_TPI_GA3P = v_TPI_GA3P*1/(k_GA3P_TPI/GA3P+1);
r_Tri = v_Tri*1/(k_GA/GA+1);
r_ATPTri = 1/(k_ATPMg/ATP+1);
r_ATPiTri = 1-eff*(1/(1+kiATPiTri/ATP));
r_ADPiTri = 1-eff*(1/(1+kiADPiTri/ADP));
r_pyr = v_PDC*1/(k_pyr/pyr+1);
r_acoaiPDC = 1-(1/(1+kiacoaPDC/acoa));
r_acoa = v_lipg*1/(k_acoa/acoa+1);
r_ATPlipg = 1/(k_ATPlipg/ATP+1);
r_FAilipg = (1-(1/(1+kiFA/FA)));
r_FA = v_tsyn*1/(k_FA/FA+1);
r_GA3Ptsyn = 1/(k_GA3P_tsyn/GA3P+1);
r_glugen = v_PEPCK*1/(k_PEPCK/pyr+1);
r_ATPglu = 1/(k_ATPglu/ATP+1);
r_GTPglu = 1/(k_GTPglu/GTP+1);
r_FAboxi = v_boxi*1/(k_boxi/FA+1);
r_aciboxi = 1-effac*(1/(1+kiacbox/acoa));
r_ATPboxi = 1/(1+k_ATPbox/ATP);
r_PPARboxi = 1-effPPAR*(1/(1+kiF1P/F1P));
r_TG = v_lipo*1/(k_TG/TG+1);
% Glucose combination
r_GK = v_GK*((Glu.^nglu)/((Glu.^nglu)+((k_glu).^nglu))) * (ATP/(ATP+k_ATPgk))*...
   (1-Hill(G6P,kig6pGK,nig6pGK));
r_G6Pase =v_G6P*G6P/(G6P+k_G6P);
h_FBP = (EX_Gcg+hFBPPFKref)/(EX_Ins+hFBPPFKref);
r_FBP = v_FBP*h_FBP*Hill(GA3P,k_FBP,1);
h_PFK = (EX_Ins+hFBPPFKref)/(EX_Gcg+hFBPPFKref);
r_PFK = v_PFK*h_PFK*Hill(G6P,k_PFK,1)*((ATP/(ATP+k_ATPpfk)))*...
   (1-(ATP/(ATP+kiatpPFK)))*(ADP/(ADP+kiadpPFK))*...
   (1-effpfk*(GA3P/(GA3P+kiga3p)));
h_PEPCK = (EX_Gcg+hPEPCKref)/(EX_Ins+hPEPCKref);
r_PEPCK = h_PEPCK*r_glugen*r_ATPglu*r_GTPglu;
h_PK = (EX_Ins+hPKref)/(EX_Gcg+hPKref);
r_PK = v_PK*h_PK*Hill(GA3P,k_GA3P_PK,1)*(ADP/(ADP+k_ADPpk))*(1-eff*(acoa/
(acoa+kiacoaPK)));
h_PDC = 1+EX_Ins/hACOAInsref-EX_Gcg/hACOAGcgref;
r_PDC = h_PDC*r_pyr*r_acoaiPDC;
h_DNL = 1+EX_Ins/hDNLInsref-EX_Gcg/hDNLGcgref;
```

```
r_DNL = h_DNL*r_acoa*r_ATPlipg*r_FAilipg;
h_boxi =1-EX_Ins/hboxiInsref+EX_Gcg/hboxiGcgref;
r_boxi = h_boxi*r_FAboxi*r_aciboxi*r_ATPboxi*r_PPARboxi;
h_TGS = 1+EX_Ins/hTGSInsref-EX_Gcg/hTGSGcgref;
r_TGS = turn(h_TGS)*r_FA*r_GA3Ptsyn;
h_Lply = 1-EX_Ins/hLplyInsref+EX_Gcg/hplyGcgref;
r_Lply = turn(h_Lply)*r_TG;

% 8. Differential Equations:
% 8.1 Plasma section
dB_Fdt = M_Fru+(R_BS*(EX_F-B_F))/R_RL;
dB_Gdt = M_Glu+(R_BS*(EX_G-B_G))/R_RL-U_G-U_DNL-U_TGS/2;
if B_G<=4500
   B_G = 4500;
elseif B_G>=12500
   B_G = 12500;
else
   dB_Gdt = M_Glu+(R_BS*(EX_G-B_G))/R_RL-U_G-U_DNL-U_TGS/2;
end
dB_Ldt = R_BS*(EX_L-B_L)/R_RL;
dB_FFAdt = R_BS*(EX_FFA-B_FFA)/R_RL-U_FFA-U_TGS+3*U_LiI+4*U_DNL;
dB_TGdt = R_BS*(EX_TG-B_TG)/R_RL+U_TGS-U_LiI;
% Plasma hormone release
dP_Insdt = (1/(tauI*rel*mins))*(Hill(Aln(BloodScale*B_G/gri),tIg,nI)) - (R_BS*(P_Ins-
EX_Ins))/R_RL;
dP_Gcgdt = (1/(tauL*rel*mins))*(Hill(Aln(gr/(BloodScale*B_G)),tLg,nL)) -
(R_BS*(P_Gcg-EX_Gcg))/R_RL;

% 8.2 Bloodstream Exchange section --- Hepatic bloodstream
dEX_Fdt = - 1/2*(T_GLUT2+T_GLUT5)*R_HE+R_BS*(B_F-EX_F);
dEX_Gdt = - T_GLUTG*R_HE+R_BS*(B_G-EX_G);
dEX_Ldt = - T_Lac*R_HE+R_BS*(B_L-EX_L);
dEX_FFAdt = - T_FA*R_HE+R_BS*(B_FFA-EX_FFA);
dEX_TGdt = - T_TG*R_HE+R_BS*(B_TG-EX_TG);
% Hepatic hormone uptake
dEX_Insdt = -(1/(tauI*mins))*(EX_Ins/MI) +R_BS*(P_Ins-EX_Ins);
dEX_Gcgdt = -(1/(tauL*mins))*(EX_Gcg/ML) +R_BS*(P_Gcg-EX_Gcg);

% 8.3 Hepatic section
dFrudt = 1/2*T_GLUT2+1/2*T_GLUT5-r_khk*r_ATPkhk;
dF1Pdt = r_khk*r_ATPkhk-r_ald;
dGludt = T_GLUTG-r_GK+r_G6Pase ;
dG6Pdt = r_GK-r_G6Pase+r_FBP-r_PFK;
dDHAPdt = r_ald-r_TPI_DHAP+r_TPI_GA3P;
dGAdt = r_ald-r_Tri*r_ATPTri*r_ATPiTri*r_ADPiTri+r_Lply;
dGA3Pdt = r_TPI_DHAP+r_Tri*r_ATPTri*r_ATPiTri*r_ADPiTri-r_PK-r_TGS-
r_TPI_GA3P+r_PEPCK-2*r_FBP+2*r_PFK;
dpyrdt = T_Lac+r_PK-r_PDC-r_PEPCK;
dacoadt = r_PDC-8*r_DNL+8*r_boxi;
dFAdt = T_FA+r_DNL-r_boxi-r_TGS+r_Lply;
```

```
dTGdt = T_TG+1/3*r_TGS-1/3*r_Lply;

% Function Outputs
dydt =[dB_Fdt; dB_Gdt; dB_Ldt; dB_FFAdt; dB_TGdt; dP_Insdt; dP_Gcgdt;...
    dEX_Fdt; dEX_Gdt; dEX_Ldt; dEX_FFAdt; dEX_TGdt; dEX_Insdt; dEX_Gcgdt;...
    dFrudt; dF1Pdt; dGludt; dG6Pdt; dDHAPdt; dGAdt; dGA3Pdt; dpyrdt; dacoadt;
dFAdt; dTGdt;...
    dlipoidt; dlipoldt; dtriidt; dtrildt];
end
```
